# Tropopause-aware IVT and IWV sharpen atmospheric river detection over North America

**DOI:** 10.1038/s41612-026-01449-x

**Published:** 2026-06-04

**Authors:** Saadoun Salimi, Taha B.M.J. Ouarda

**Affiliations:** Canada Research Chair in Statistical Hydro-Climatology, Institut National de la Recherche Scientifique, Centre Eau Terre Environnement, INRS-ETE, Québec, QC Canada

**Keywords:** Climate sciences, Environmental sciences, Hydrology

## Abstract

Atmospheric rivers are among the most consequential moisture-transport systems shaping flood risk, hydroclimatic extremes, and water cycles across North America. Standard IVT/IWV threshold methods, however, fail to capture slender ARs or accurately gauge intensity due to neglected upper-tropospheric thermodynamics. Here, using ERA5 reanalysis (1984–2024), we modified IVT and IWV by incorporating a tropopause-aware thermodynamic weighting function calibrated with radiosonde profiles. Validation against radiosondes and SSMIS imagery confirms that the modified indices improve AR intensity and geometry estimation by 8.7% and narrow-filament detection by 12%. Four-decade seasonal trends reveal robust AR increases in spring, summer, and winter over eastern North America, and in winter and summer over the west. Furthermore, the modified IVT and IWV better revealed the dominant role of remote drivers in North American AR activity. Accordingly, stepwise regression findings showed that ENSO, PNA, PDO, and NAO explain more than 45% of regional AR variance. These findings emphasize the urgent need to embed upper-tropospheric thermodynamics in AR detection and their precise spatiotemporal activity for improved flood forecasting and climate projection.

## Introduction

A key process driving extreme precipitation and long-range vapor transport is the atmospheric river (AR). These features appear as transient, filamentary corridors of water vapor that channel tropical moisture into higher latitudes^1^. At any instant, a typical AR conveys more than twice the volume of the Amazon River, the planet’s most extensive fluvial system^2,3^. Upon impinging on mountainous coastlines, ARs force intense orographic uplift, producing heavy rainfall at lower elevations and snowfall at higher elevations^4^. ARs exert a controlling influence on regional hydroclimate, most notably along the western North America^5–7^. Along North America’s Pacific margin, powerful low-level winds trigger marked dynamic and thermodynamic adjustments in the upper ocean, which climate change intensifies^8^. Concurrently, ocean dynamics exert substantial control over surface warming, exposing a tight ocean–atmosphere linkage during severe AR events^9^. From a climatological perspective, ARs supply up to 65% of annual precipitation in northern California and up to 40% farther south^10^. The most severe impacts concentrate along the west coast of North America (NA)^11–13^, western Europe^14^, and select northwest African coastal zones^15^.

Forecasting AR occurrence and its precipitation remains difficult due to the interplay of Teleconnection indices across timescales like ENSO, Pacific Decadal Oscillation (PDO), Madden–Julian Oscillation (MJO)^16^, PNA, and Arctic Oscillation (AO)^17–20^. Teleconnections describe statistically significant correlations between distant atmospheric circulation patterns and provide a framework for characterizing the transport of heat, energy, and moisture as well as regional climate and ecosystem changes^21–27^. Lee and Ouarda (2025) developed a novel stochastic simulation model using remote drivers to generate synthetic series linked to climate signals^28^. Extensive research throughout the 20th and 21st centuries has relied predominantly on classical statistical-synoptic approaches. Key studies have shown, for example, negative correlations between polar and mid-latitude sea-level pressure^29^, ENSO-driven wind and temperature anomalies in Kuwait and Australia^30,31^, El Niño/La Niña influence on the Arctic Oscillation via ±PNA phases^32^, and the role of multiple indices, including Eurasian Zonal Circulation Index (EZI), Niño 3.4, and Indian Ocean Dipole (IOD) in precipitation, temperature, and runoff variability in Asia^33,34^.

Since rainfall responds to large-scale teleconnection patterns, ARs interact with these modes^35^. Central Pacific El Niño events displace the Aleutian Low equatorward, boosting AR activity across the northeast Pacific, while La Niña reinforces anticyclonic circulation that suppresses ARs in the eastern basin^36,37^. Cool-season AR precipitation in the Sierra Nevada, however, shows no robust ENSO signature, unlike non-AR rainfall, which increases under El Niño-like conditions^38^. Instead, near-neutral ENSO paired with a positive PDO favors Pineapple Express-style ARs and directs tropical moisture plumes toward the U.S. West Coast^39,40^. Over multi-decadal timescales, the PDO, not ENSO, emerges as the primary control on AR landfall frequency along western North America^10^, although combined ENSO–PDO phases dictate the latitude and meridional alignment of landfalling ARs in northern California^41^. These subtle shifts in storm-track geometry and AR orientation, rather than frequency changes per se, ultimately explain the observed ENSO imprint on regional precipitation^41–44^. On shorter timescales, sub-seasonal modes such as the MJO^44^ and Pacific–Japan pattern frequently override ENSO influence^45–47^, conditional forecast skill extends to 7–10 days under specific ENSO/ PNA configurations^48^, southwestern U.S. ARs can amplify or obscure canonical ENSO signals^49^, and extreme streamflow responds to a broader suite of modes^31^. Recent AR trends along both U.S. coasts reflect large-scale Pacific reorganization and thermodynamic moistening^50–52^. Dynamically, Jet streams, particularly the polar-front jet generated by meridional thermal gradients, dominate the dynamical processes that give rise to ARs^53–55^. Salimi and Ouarda (2025) found that in western NA, these jets have climbed to higher altitudes during autumn and winter^56,57^. Their results also revealed consistent positive temperature trends between 20° and 55° N across the continent, as well as North Pacific trends that likely exert a strong influence on jet velocities, AR morphology, and the efficacy of detection algorithms^56^. A variety of methods have been deployed to identify ARs, but integrated vapor transport (IVT) and integrated water vapor (IWV) remain the most widely adopted. Over the North Pacific, Dong et al. (2025) utilized CMIP5 and CMIP6 ensembles to simulate ARs, which proved markedly shorter and narrower than those catalogued in the ERA5 reanalysis^58^. The discrepancy probably reflects the tendency of the Tempest Extremes (TE) algorithm^59^ when applied to coarser model grids, to render ARs smoother and less elongated at their downstream ends. The same study showed that the Mundhenk method systematically yields ARs that are larger in areal extent, width, and length than those from TE. Despite substantial differences among detection schemes, the ARTMIP intercomparison indicated broadly comparable performance, with individual results clustering near the multi-method average^60^. Globally, IVT bias reduction tracks closely with low-level wind bias improvement, yet lags far behind the gains achieved for total precipitable water^61^. ERA5-derived IWV and IVT, however, can underestimate and overestimate proper AR moisture, particularly in narrow filaments, by up to 21%^62,63^. This systematic shortfall led us to incorporate upper-tropospheric thermodynamic effects, specifically those focused on the tropopause, directly into the IWV and IVT formulations to enhance detection fidelity. The approach is grounded in Salimi and Ouarda (2025), who documented coupled tropopause warming and jet-stream displacement^56^.

Given the pronounced spatiotemporal variability of ARs across NA, their well-established links to remote climate drivers, and the diagnostic importance of IWV and IVT, this study seeks to refine AR detection metrics by incorporating upper-tropospheric thermodynamic information to improve the identification of narrow AR filaments and their embedded vapor intensity features for which standard IWV and IVT often exhibit limited skill^64,65^. Advancing these diagnostics is particularly essential because ARs are among North America’s most impactful hydro-meteorological phenomena, directly affecting water resources, reservoir operations, and flood risk. The lack of precise continent-wide spatiotemporal AR analysis heightens this need. Accordingly, the methodological developments presented here aim to reduce existing detection biases, enhance physical realism, and provide more reliable tools for future AR monitoring and prediction.

## Results

### Validation of modified indices against radiosonde data

**Fig. 1:**
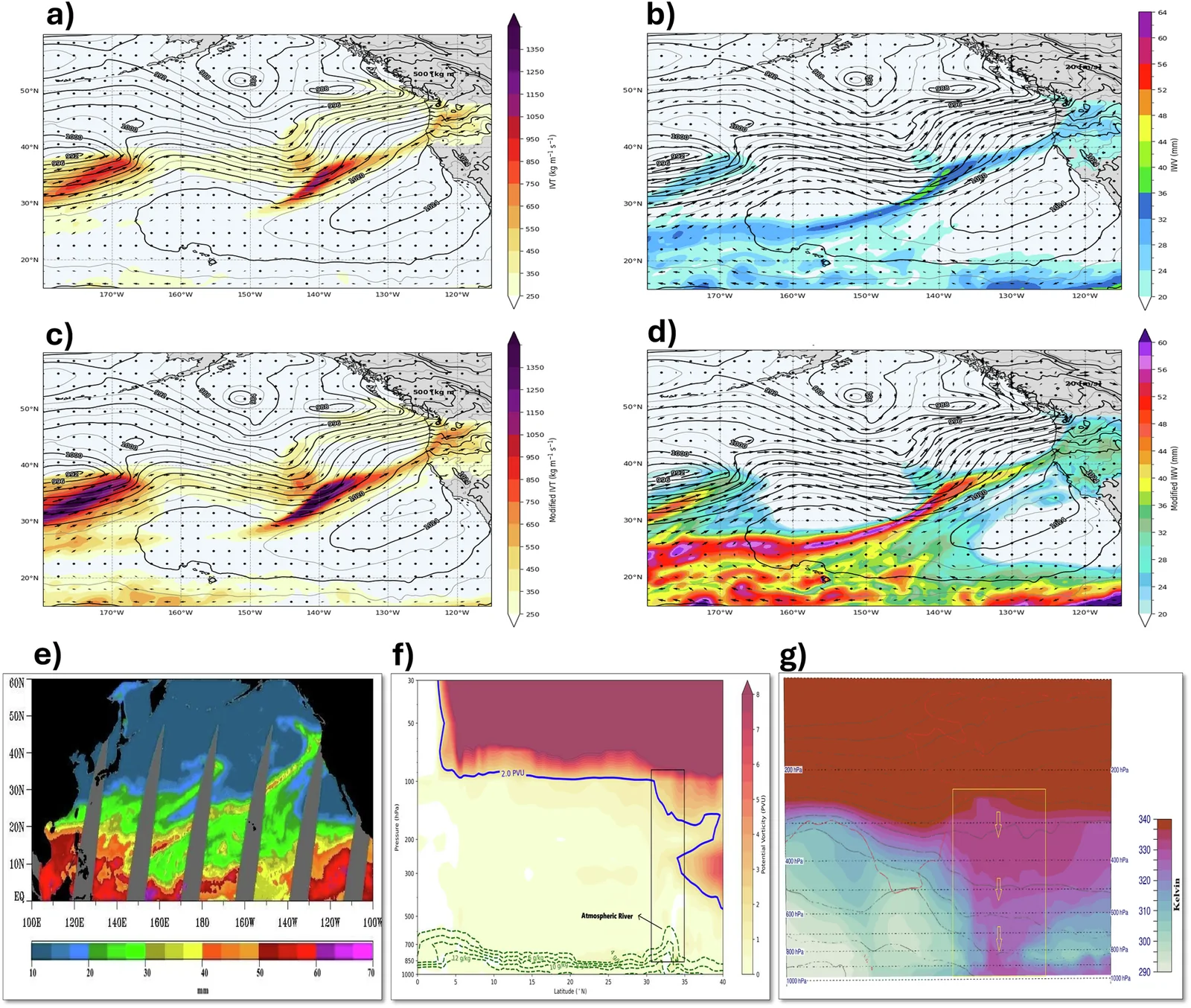
Comparison of standard and modified indices for AR detection. Maps show an atmospheric river (AR) event over the North Pacific on February 23, 2025, identified using standard Integrated Vapor Transport (IVT; **a**) and Integrated Water Vapor (IWV; **b**) indices, compared with the thermodynamically modified IVT (**c**) and modified IWV (**d**) formulations. The modified indices (**c**–**d**) illustrate enhanced detection of narrow features and the physical structure of the moisture plume. Satellite imagery in (**e**) displays the corresponding water vapor observed by SSMIS for visual validation of the event geometry. **f**, **g** Provide vertical cross-sectional analysis along the AR core: (**f**) depicts the coupling between the stratospheric potential vorticity (PV) intrusion, indicated by the solid blue 2.0 PVU dynamic tropopause line, and the low-level specific humidity core shown by green dashed contours; (**g**) illustrates the vertical distribution of equivalent potential temperature (Kelvin, see color bar) in relation to the dynamic tropopause above the AR. Purple and orange shading in (**a**–**d**) represent magnitudes of moisture transport and content, respectively, while black contours indicate sea-level pressure (hPa).

In this study, the two standard indices commonly used for AR detection, IVT and IWV, were first applied to extract 20 narrow and detection-sensitive AR events that passed over the Hawaiian Islands (Table 1). One such event, from 23 February 2025, is shown in Fig. 1 to illustrate the diagnostic difficulty. Although high atmospheric moisture was confirmed on that day by reanalysis of data and satellite imagery, the actual moisture content was not adequately captured by the conventional indices (Fig. 1a, b) due to their inherent limitations^64,65^. The modified IVT and IWV indices were therefore employed, with results presented in Fig. 1c, 1d. As observed, the revised indices delineate the AR in question with substantially greater accuracy and resolution.

**Table 1:** Comparison of observed and modified IVT and IWV values from ERA5, and radiosonde formulation for 20 AR events over Hawaii

Case	IVT-standard	IVT-modified	IVT-radiosonde	IWV-standard	IWV-modified	IWV-radiosonde	Date
**1**	415	460	475	31.8	34.1	36.9	30/01/2013
**2**	482	515	540	33.7	36.3	38.6	08/12/2017
**3**	389	420	445	28.5	30.8	33.1	05/04/2005
**4**	505	550	563	36.1	38.7	41.5	19/01/2012
**5**	572	602	635	38.2	41.5	44.6	23/01/2017
**6**	423	455	485	31.0	33.2	36.2	22/01/2018
**7**	601	650	668	41.0	44.8	47.3	16/12/2022
**8**	479	520	548	34.1	37.6	39.8	13/12/2015
**9**	523	561	587	36.2	39.1	42.4	11/12/2010
**10**	400	438	462	29.4	32.2	35.1	21/10/2005
**11**	472	510	534	33.1	36.4	38.8	02/01/2013
**12**	529	565	592	37.3	40.2	43.7	16/01/2006
**13**	545	580	609	38.4	41.1	44.9	20/10/2015
**14**	362	395	421	26.8	29.3	31.6	09/03/2016
**15**	622	672	685	42.2	45.3	48.1	11/01/2009
**16**	427	458	489	30.9	33.5	36.6	07/02/2018
**17**	490	523	556	34.8	37.8	40.6	07/12/2018
**18**	541	579	605	38.1	40.7	44.1	13/10/2004
**19**	507	545	572	35.4	38.5	41.3	31/01/2020
**20**	386	418	451	28.2	31.0	33.7	15/02/2007

Further validation was conducted using actual atmospheric vapor fields derived from the SSMIS, which effectively depict the accurate moisture volume^66^ (Fig. 1e). Both the total moisture content and the spatial dimensions, length, and width of the AR were thus represented with higher fidelity by the modified IVT and IWV indices compared to their standard counterparts. To bolster this assessment statistically, all 20 AR events were subsequently examined. To elucidate the physical mechanisms underpinning these improvements, vertical cross-sections of potential vorticity (PV) and equivalent potential temperature (EQPT) were examined (Fig. 1f, 1g). Figure 1f reveals a pronounced stratospheric PV intrusion, delineated by the 2.0 PVU dynamic tropopause contour, descending toward the mid-troposphere between 30°N and 35°N. This downward displacement of the tropopause aligns precisely with the localized moisture core of the atmospheric river (AR; green dashed contours), pointing to a tight dynamical coupling between upper-level and lower-tropospheric processes.

The corresponding vertical distribution of EQPT in Fig. 1g displays a clear ridge-like structure, or thermal bulge, within the same latitude band. This pattern signals localized destabilization accompanied by adiabatic warming, most likely amplified by latent-heat release in the AR’s^67–69^ ascending airstream. The juxtaposition of the depressed tropopause with the moisture-laden tropospheric air establishes sharp thermal and pressure gradients at the interface^70,71^. By explicitly incorporating these upper-level thermodynamic anomalies into the revised formulations, the analysis captured critical stratospheric–tropospheric interactions that conventional indices systematically fail to resolve, thereby yielding the markedly improved detection fidelity demonstrated in this study.

Table 1 presents a case-by-case comparison between the observed radiosonde data and two diagnostic indices (standard and modified IWV and IVT) for 20 selected AR events over Hawaii. These events were randomly selected from a larger pool of ARs transitioning over the Hawaiian Islands to ensure a representative sample for validating our methodology. The observed data from radiosondes were used to calculate reference IVT and IWV. The choice of Hawaii as the focal point for this validation is strategic^72^; the Hawaiian radiosonde station offers one of the most advantageous geographical positions globally for monitoring the longitudinal evolution and vertical structure of Atmospheric Rivers^73^. The standard ERA5 generally underestimates both IVT and IWV compared to the observations, reflecting the typical smoothing bias of coarse-resolution reanalysis. In contrast, the modified IWT and IVT, which incorporate vertical temperature gradient weighting (W∇Tₜᵣₒₚ), yield higher IVT and IWV values that align more closely with the observed radiosonde profiles. This enhancement is especially evident during intense and narrow AR events, indicating that the thermodynamic adjustment improves moisture-transport representation in the lower troposphere.

**Fig. 2:**
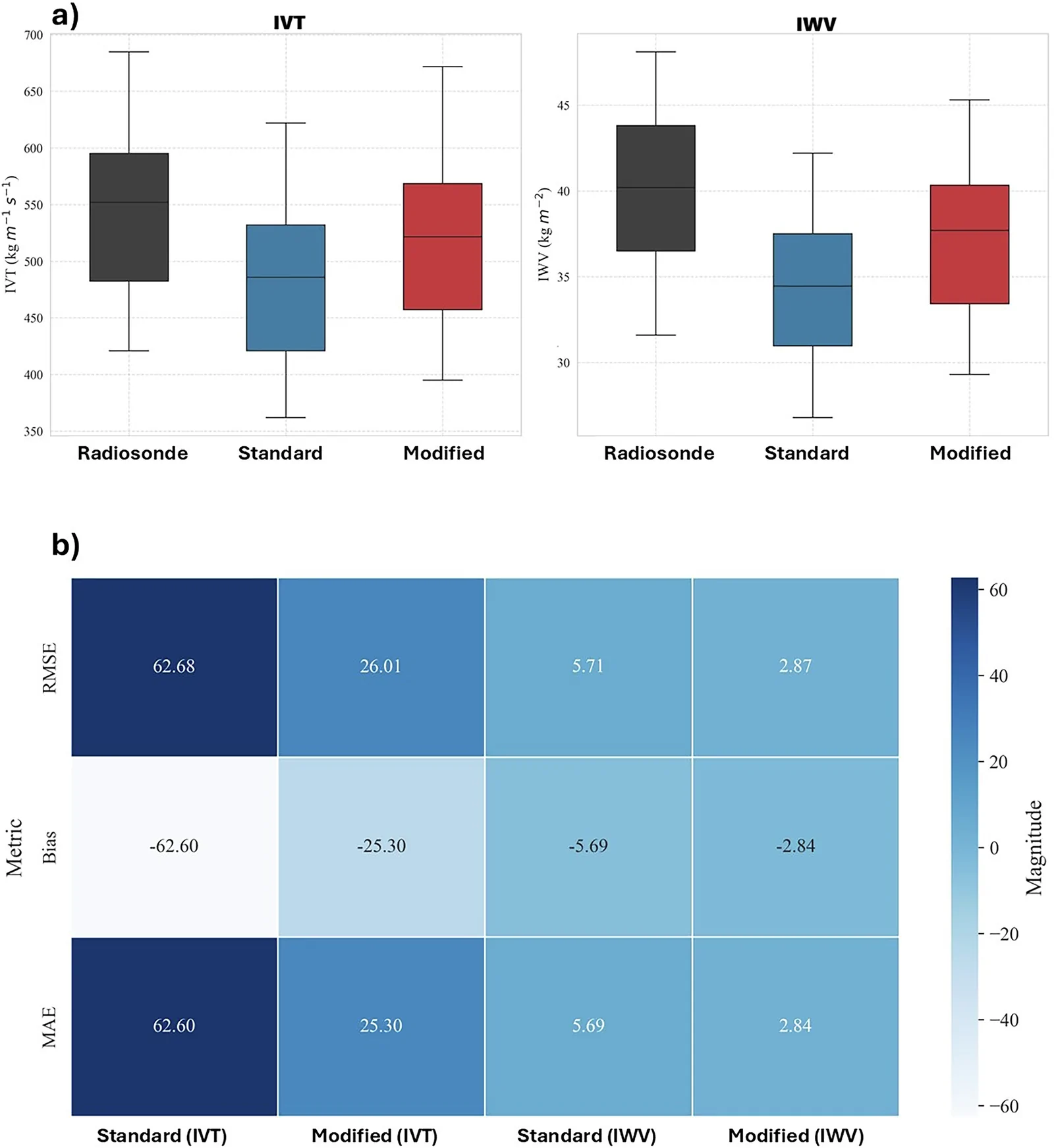
Statistical evaluation of modified metrics against radiosondes. **a** Boxplots compare IVT and IWV distributions across Radiosonde (grey), Standard (blue), and Modified (red) configurations. Central horizontal lines represent the median, while whiskers extend to the minimum and maximum values. The heatmap in (**b**) summarizes key error metrics including RMSE, Bias, and MAE for both standard and modified configurations. Shading in the heatmap represents the magnitude of each metric, with the color bar on the right indicating the scale from −60 to +60. The results demonstrate that the modified indices (light blue columns in **b**) yield significantly lower RMSE and MAE values compared to the standard versions (dark blue columns).

Table 2 and Fig. 2 summarize the statistical performance metrics derived from the 20 event comparisons shown in Table 1. As expected from the validation design, the mean modified IVT (520.8 kg m⁻¹ s⁻¹) is closer to the observed radiosonde value (546.1 kg m⁻¹ s⁻¹) than the standard IWV and IVT (483.5 kg m⁻¹ s⁻¹). A nearly identical pattern emerges for IWV, where values improved from 34.26 mm (standard) to 37.1 mm (modified), now approaching the observed mean of 39.9 mm. Notably, the Relative Improvement is 8.7%, the RMSE and MAE decreased by approximately 7%, while the correlation coefficient 0.99, indicating a more realistic coupling between the thermodynamic and dynamic components of moisture transport. This gain is especially evident in narrow filaments. Given that the characteristic width of ARs typically ranges between 500 and 1000 km^74,75^, we specifically defined events with an AR width of approximately 500 km as “narrow” for this analysis. In these cases, the detection accuracy improved from 79% to 91%. These results collectively confirm that incorporating vertical thermal gradients enhances the physical realism of IVT and IWV estimates in ERA5-based analyses.

**Table 2:** Statistical comparison of standard IVT and IWV, modified IWV&IVT, and radiosonde

Metric	Standard IWV and IVT	Modified IWV and IVT	Radiosonde
**Mean IVT (kg·m⁻¹·s⁻¹)**	483.5	520.8	546.1
**Mean IWV (mm)**	34.2	37.1	39.9
**RMSE**	IVT: 62.6 IWV: 5.7	IVT: 26 IWV: 2.8	-
**Bias**	IVT: -62.6 IWV: -5.6	IVT: -25.3 IWV: -2.8	-
**MAE**	IVT: 62.6 IWV: 5.6	IVT: 25.3 IWV: 2.8	-
**Correlation (***r***)**	IVT: 0.998 IWV: 0.997	IVT: 0.997 IWV: 0.996	1
**Standard Deviation (***σ***)**	IVT: 72.2 IWV: 4.2	IVT: 75.3 IWV: 4.4	IVT: 73.6 IWV: 4.6
**Relative Improvement (%)**	—	≈ 8.7%	-
**Detection Accuracy of Narrow ARs (%)**	79	91	-

### AR geometry and intensity capturing performance

**Fig. 3:**
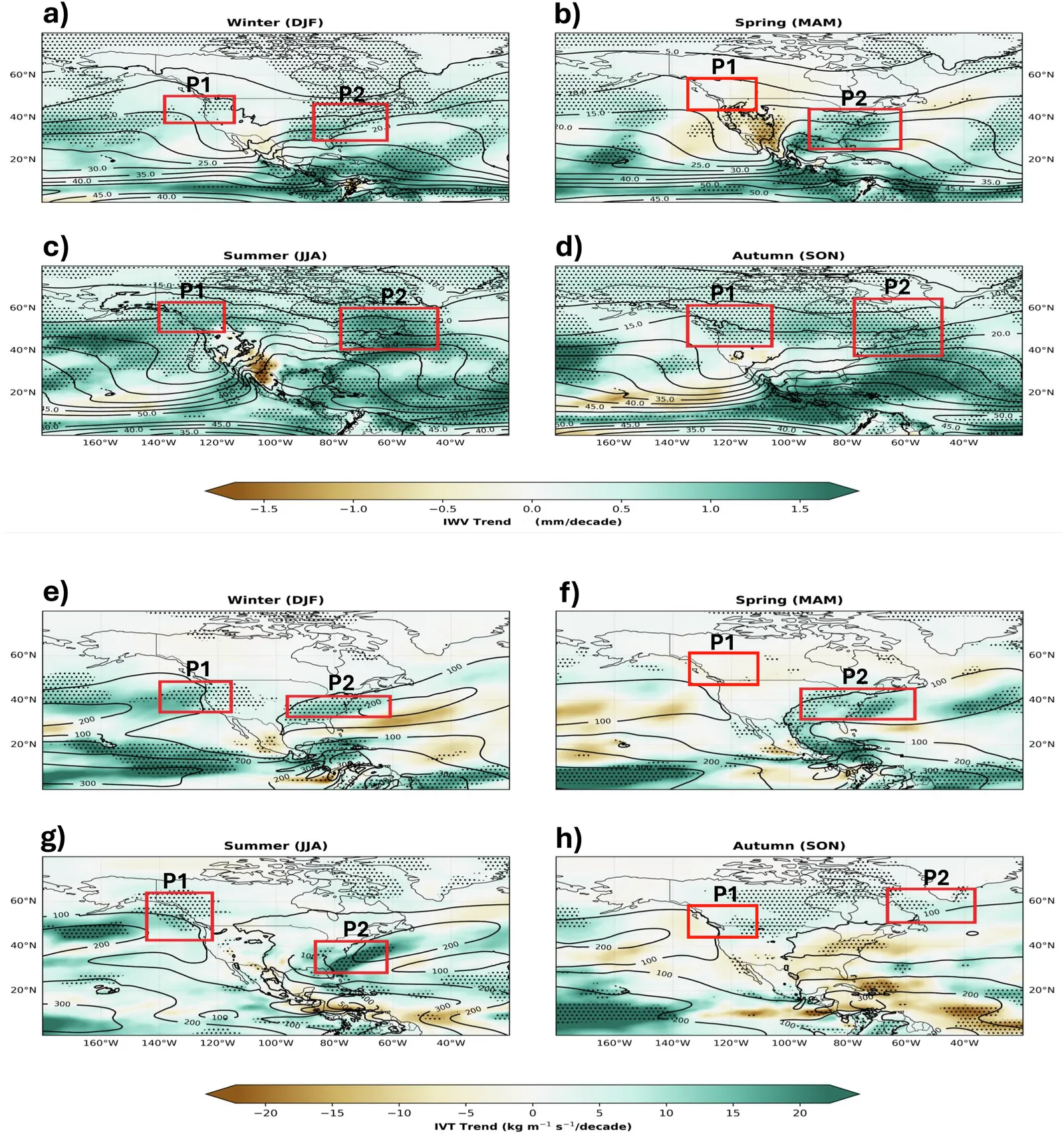
Spatial distribution of seasonal AR trends over North America. The maps illustrate decadal linear trends derived from the thermodynamically modified Integrated Water Vapor (IWV; **a**–**d**) and Integrated Vapor Transport (IVT; panels **e**–**h**) indices. Each panel represents a specific season: winter (**a**, **e**), spring (**b**, **f**), summer (**c**, **g**), and autumn (**d**, **h**). Shading denotes the magnitude of the trend, with teal representing positive trends and brown representing negative trends as indicated by the respective color bars (mm/decade for IWV and kg m⁻¹ s⁻¹/decade for IVT). Black stippling indicates regions where trends are statistically significant at the 95% confidence level. Red rectangles, labeled P1 and P2, demarcate the western and eastern North American corridors, respectively, which serve as the basis for the detailed frequency and intensity analyses in subsequent figures.

The spatial distribution of seasonal AR frequency trends, as derived from the modified IWV indices, revealed predominantly positive trends across NA. Statistically significant increases were observed in spring and summer over eastern NA and in winter and summer over the western sector. In all seasons, the areal extent of significant trends was greater in the east than in the west. The southern and southwestern regions, by contrast, exhibited decreases in AR frequency based on the modified IWV indices during most seasons, with the strongest and most extensive declines occurring in the southwest during spring. These negative trends, however, were generally not statistically significant. This regional contrast in the Southwest is consistent with recent climatological evidence of unexpected atmospheric drying and significant increases in Vapor Pressure Deficit (VPD) in the interior Southwest^76^. These localized anomalies, likely driven by shifts in large-scale circulation patterns, explain the suppression of moisture availability for vapor streams in this sector despite the broader continental moistening trend. Red dots and boxes in Fig. 3a–d indicate the spatial extent and significance of seasonal AR trends.

Linear trend analysis based on the modified IVT indices similarly indicated widespread positive trends in AR frequency across most seasons and regions. Significant increases were detected in autumn, spring, and winter over eastern NA, as well as in winter and summer over western and northwestern areas. Southern regions exhibited a decline in AR frequency in nearly all seasons, with additional decreases in the southeast during autumn. As with modified IWV, these negative trends were largely non-significant. The areal coverage of significant trends remained greater in the east than in the west across all seasons. Moreover, the magnitude of intensification derived from the modified IVT indices exceeded the moistening trends of the modified IWV indices (Fig. 3e–h).

### Seasonal AR trends across North America (1984–2024)

**Fig. 4:**
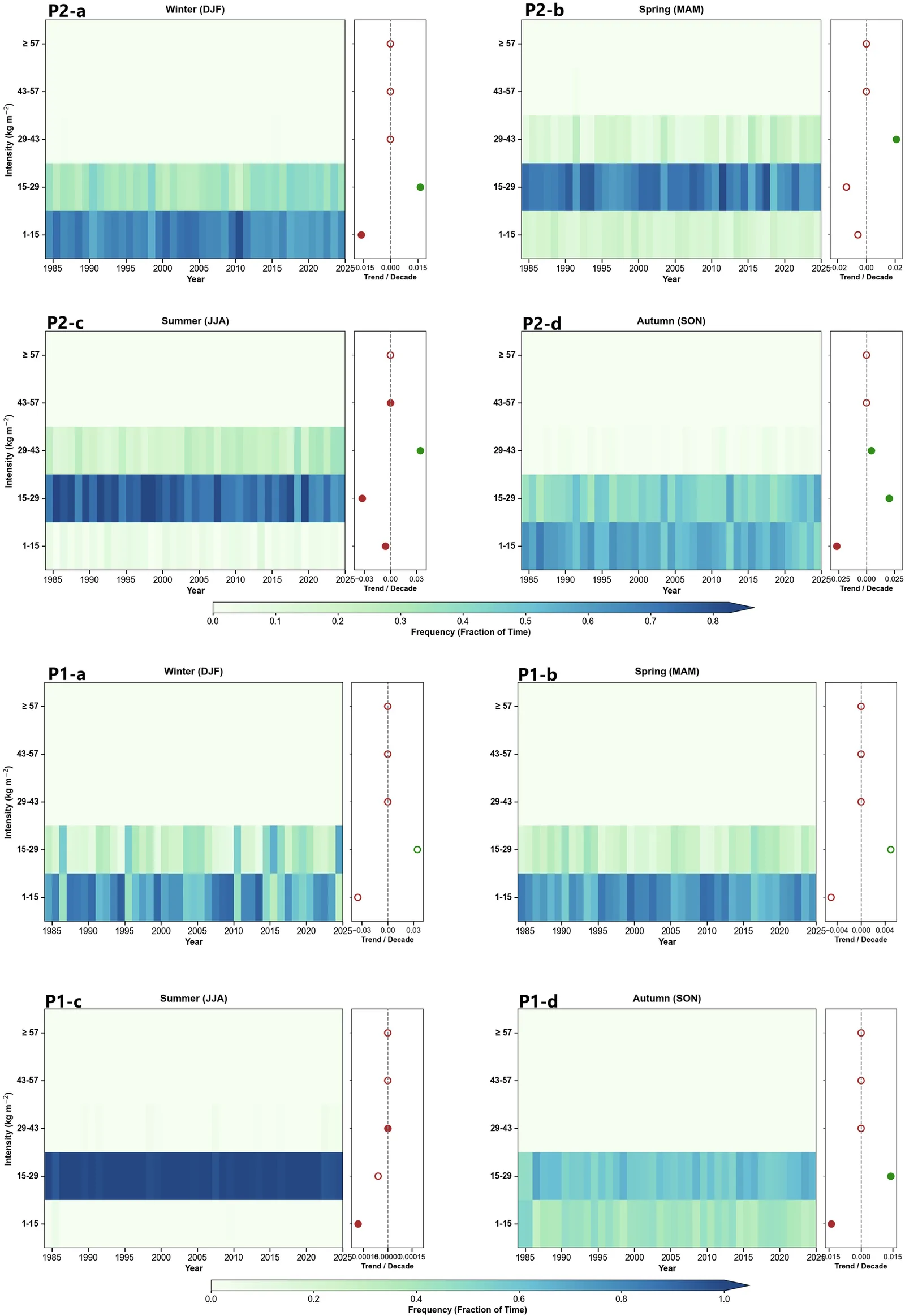
Seasonal IWV frequency and trends in primary AR corridors. Seasonal frequency and decadal trends of AR activity were derived from the modified IWV index during 1984–2024. The analysis corresponds to the western (**P1**) and eastern (**P2**) corridors highlighted in Fig. 3a–d. (**P1**-**a**–**d**) and (**P2**-**a**–**d**) illustrate the relative frequency of ARs across IWV intensity categories (color shading) and their associated decadal trend slopes (dots at right). Positive (green) and negative (red) markers indicate increasing or decreasing frequencies, respectively, for each season (DJF, MAM, JJA, SON), revealing distinct regional and seasonal contrasts in AR intensity evolution.

Following the extraction of ARs using the thermodynamically modified IVT and IWV indices, the seasonal intensity distribution and temporal trends of AR occurrences were analyzed across the eastern and western regions of NA from 1984 to 2024 using linear regression. According to the modified IWV results, the highest AR intensities were recorded in autumn, ranging from 15 to 57 mm on average, followed by winter, with intensities of 1 to 43 mm. The lowest intensity levels were observed during spring (1–29 mm) and summer (1–15 mm) throughout the analysis period. On an interannual basis, the maximum seasonal intensities occurred in winter 2011, spring 2018, summer 1997, and autumn 1987. The seasonal trend analysis reveals a general increase in moisture content across all seasons over the four decades, with linear regression slopes ranging from 0.1 to 0.8; the most substantial increases occur during spring and summer (Fig. 4P2-a-d). In the western portion of NA, the results similarly showed that the highest AR intensities were concentrated in autumn and winter, ranging from 15 to 57 mm. At the same time, spring and summer exhibited lower intensities, averaging 1 to 29 mm during the analysis period. On an annual basis, the strongest AR intensities occurred in winter 1994 and 2014, spring 1999, summer 2021, and autumn 1987. The linear trend analysis further indicates that AR intensity in the western regions has also increased over time, with slopes ranging from 0.2 to 0.9; the most pronounced rises are observed during summer and winter (Fig. 4P1-a-d).

**Fig. 5:**
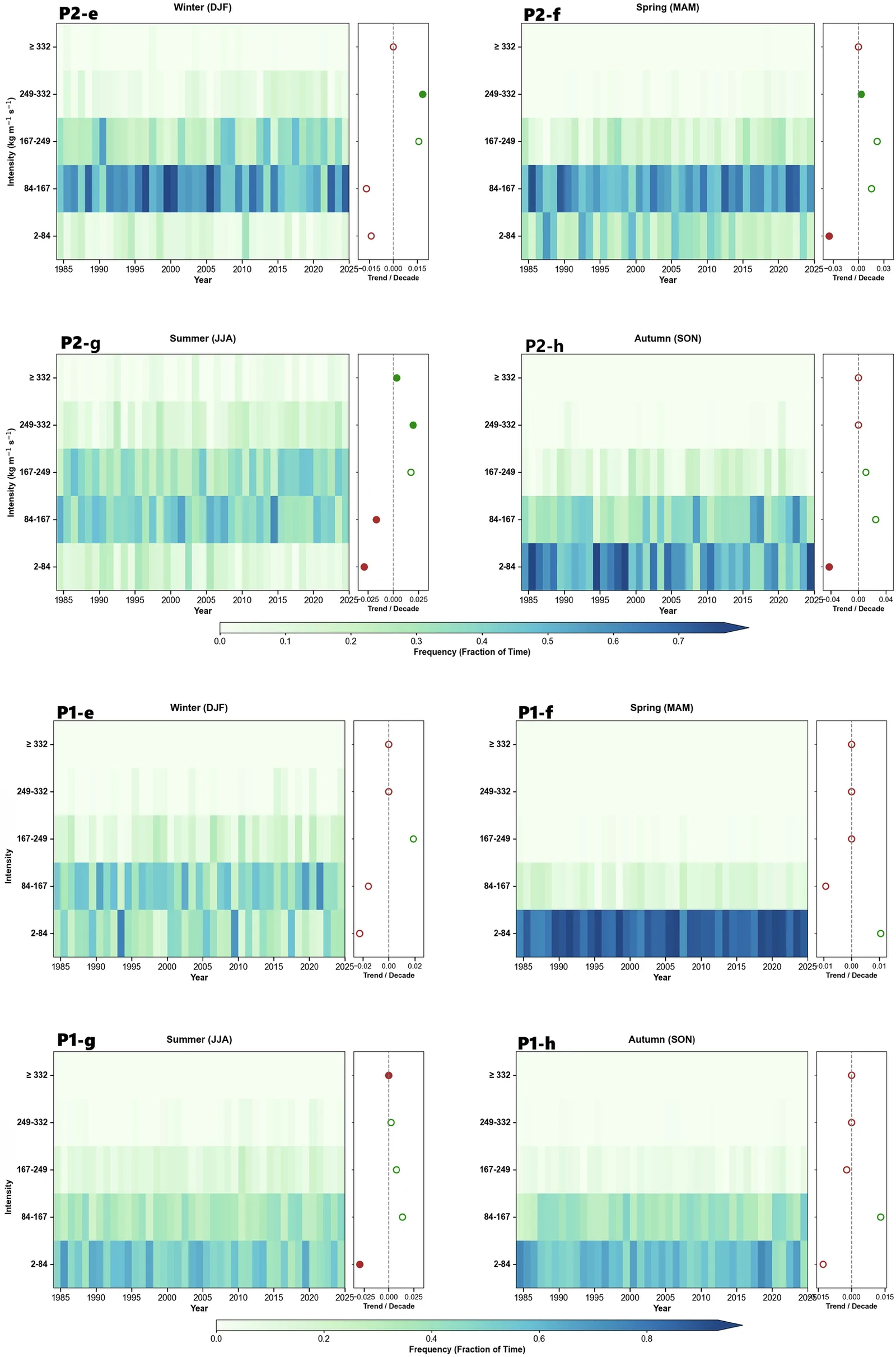
Seasonal IVT frequency and trends in primary AR corridors. Seasonal frequency and decadal trends of AR activity were derived from the modified IVT index during 1984–2024. The analysis corresponds to the western (**P1**) and eastern (**P2**) corridors highlighted in Fig. 3e–h. (**P1**-**e**–**h**) and (**P2**-**e**–**h**) Illustrate the relative frequency of ARs across IVT intensity categories (color shading) and their associated decadal trend slopes (dots at right). Positive (green) and negative (red) markers indicate increasing or decreasing frequencies, respectively, for each season (DJF, MAM, JJA, SON), revealing distinct regional and seasonal contrasts in AR intensity evolution.

The analysis of AR intensity based on the thermodynamically modified IVT over the eastern region demonstrated that the strongest moisture transport occurred during autumn and winter, with intensity values ranging from 84 to 332 kg m⁻¹ s⁻¹ throughout the four decades. The lowest intensities were observed during spring and summer, ranging from 2 to 84 kg m⁻¹ s⁻¹. On an interannual scale, the most increased IVT intensities were recorded in winter 1997 and 1999, spring 1986 and 2013, summer 2014, and autumn 1986, 1995, and 1999. The seasonal trend analysis exhibited consistent IVT intensification across all seasons in the eastern region during the period. The linear regression slopes ranged from 0.1 to 0.7, with the most noticeable increases occurring during winter and autumn (Fig. 5P2-e-h). In the western portion of NA, the maximum IVT intensities were also concentrated in autumn and winter, with average values ranging from 167 to 332 kg m⁻¹ s⁻¹. Conversely, spring and summer displayed lower intensities, ranging from 2 to 84 kg m⁻¹ s⁻¹. On an interannual basis, the strongest intensities were recorded in winter 1993 and 2009, spring 2007 and 2018, summer 1997 and 2019, and autumn 1984 and 2019. This alignment between IWV and IVT intensity distributions reinforces the physical coherence of our modified framework in capturing the seasonal peak of AR activity. Similar to the eastern region, western NA exhibited a positive trend in IVT intensity across all seasons, with linear regression slopes ranging from 0.2 to 0.9; the strongest increase occurred during autumn and spring (Fig. 5P1-e-h).

Seasonal and regional trends in AR activity were derived from the modified IVT and IWV indices across the entire 1984–2024 period. Trends in AR frequency were examined, and increases in both eastern and western NA were found to be primarily attributable to strong and exceptional AR events, while in contrast, reductions in AR frequency were observed across all intensity categories in southern NA. However, these negative trends were generally weak and did not achieve statistical significance. These results are consistent with the findings reported by Dong et al. (2025), which demonstrate that shifts in AR frequency and intensity profoundly affect associated precipitation regimes. Changes in mean precipitation were indicated to closely mirror variations in AR frequency and intensity, thereby contributing to the evolution of wet-dry patterns across NA. The intensification of AR-related extreme precipitation along both the eastern and western coasts of NA was also confirmed through an analysis of observational records^50^.

### Influence of climate drivers on regional AR activity

Given that spatiotemporal variations in AR frequency and intensity are directly influenced by large-scale atmospheric and oceanic oscillations^77,78^, relationships between the modified IVT and IWV indices and major teleconnection patterns were subsequently investigated. Seasonal correlation analysis was therefore performed between the modified IVT and IWV and principal remote drivers, with spatial correlation fields investigated across the study domain.

Fig. S1 illustrates the seasonal correlation of 12 large-scale climate modes, including AO, NAO, PNA, PDO, MJO, CENSO, MEIv2, and ENSO phases (Niño 1 + 2, Niño 3, Niño 3.4, Niño 4, and Oceanic Niño indices ONI), with modified IVT and IWV. The most substantial correlations, reaching magnitudes of up to ±0.6, depending on the season, were identified between AR activity and the AO, NAO, PNA, PDO, ENSO, and Niño 3.4 indices. The highest correlations were consistently observed in eastern NA, followed by the western and southern sectors. Nevertheless, the dominant teleconnections were found to differ substantially between regions. As depicted in Fig. S1, AR frequency in the western domain was most strongly modulated by ENSO and AO. In contrast, in the eastern part of the study area, PNA was established as the primary driver, with ENSO assuming a secondary role (Fig. S1).

**Fig. 6:**
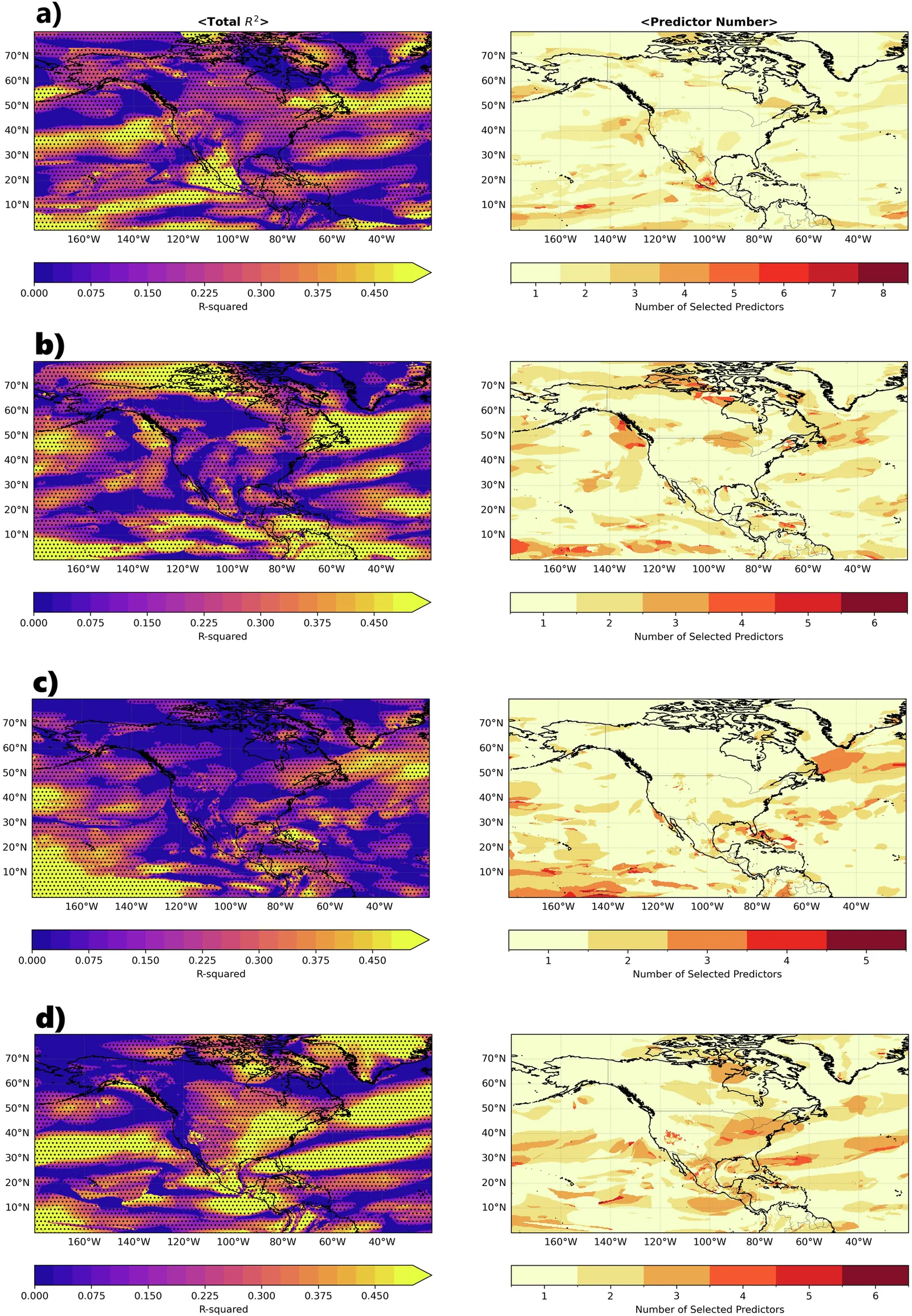
Stepwise multiple linear regression analysis of modified IVT. Stepwise multiple linear regression analysis was performed on the modified IVT across four seasons: (**a**) spring, (**b**) summer, (**c**) autumn, and (**d**) winter. The left panels illustrate the coefficient of determination (*R*²) between the observed and reconstructed atmospheric river frequency derived from the regression model. The right panels display the number of selected teleconnection predictors (count shown in color bar) that contribute significantly to each grid cell. Black stippling (dots) indicates regions where R² values are statistically significant at the 95% confidence level. These results reveal the dominant teleconnection controls and seasonal contrasts governing atmospheric river variability across North America.

**Fig. 7:**
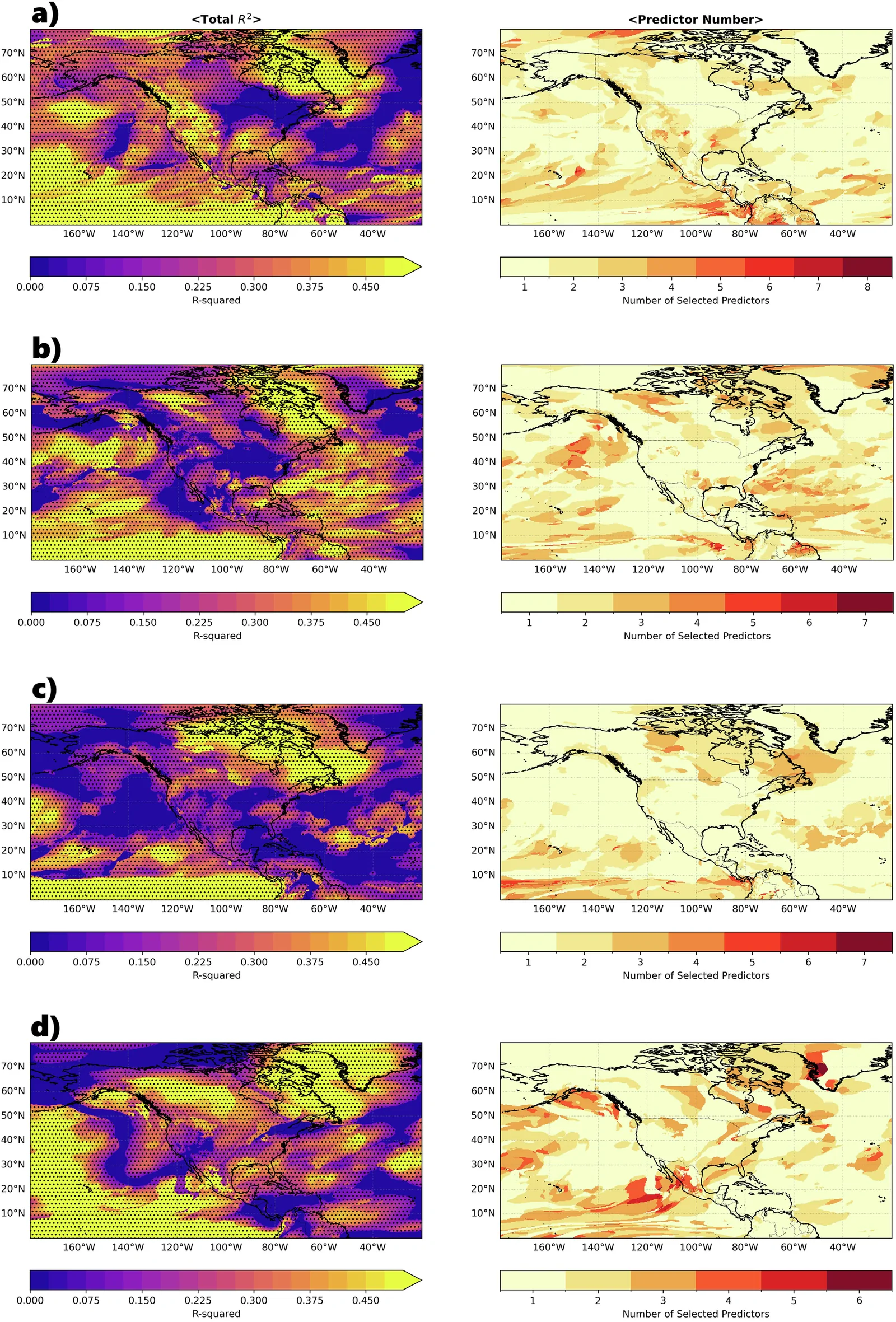
Stepwise multiple linear regression analysis of modified IWV. Stepwise multiple linear regression analysis was conducted on the modified IWV across four seasons: (**a**) spring, (**b**) summer, (**c**) autumn, and (**d**) winter. The left panels illustrate the coefficient of determination (*R*²) between observed and reconstructed atmospheric river frequency derived from the stepwise regression model. The right panels display the number of selected teleconnection predictors (count indicated in the color bar) that contribute significantly to each grid cell. Black stippling (dots) indicates regions where R² values are statistically significant at the 95% confidence level. Collectively, these results reveal the dominant teleconnection controls and seasonal contrasts governing atmospheric river variability across North America.

To identify the most influential teleconnection patterns modulating ARs and to evaluate their regional disparities, a stepwise multiple linear regression (MLR) was employed. Our analysis reveals that across most of the domain, two to three dominant teleconnection modes consistently emerge as primary drivers of AR frequency, with the most decisive influence observed along the eastern and western margins of North America. These dominant modes shift geographically and seasonally, impacting moisture content (IWV) and vapor transport (IVT) pathways differently, with a more pronounced teleconnection influence concentrated in the eastern portion of North America. This suggests distinct regional differences in how large-scale atmospheric patterns modulate moisture convergence and transport dynamics. Winter represents the period of maximum atmospheric coupling for both IWV and IVT-based AR metrics, when teleconnection patterns exert the strongest control over AR characteristics, particularly over eastern North America, Greenland, and Mexico. Spring acts as a transitional phase characterized by heightened complexity, as multiple teleconnection modes interact to influence AR activity over Mexico. Conversely, summer exhibits the weakest teleconnection signal, implying that localized thermodynamic processes may dominate during the warm months. Autumn reflects intermediate complexity with moderate teleconnection control. The spatial concentration of robust teleconnection influence in eastern North America and its alignment along the North American jet-stream corridor indicates preferential pathways through which remote climate signals (such as tropical Pacific SST anomalies and high-latitude atmospheric oscillations) propagate to modulate moisture advection and AR genesis. Detailed seasonal predictive relationships and regional variations are summarized in Table 3, while the spatial distribution of this influence is illustrated in Figs. 6 and 7.

**Table 3:** Stepwise regression analysis of IVT and IWV – seasonal summary (1984–2024)

Season	Metric	Max *R*²	Region of Highest *R*² (> 0.35)	Mean *R*²	Max of Predictors	Region of Highest Predictor Count (≥ 5)
Spring (MAM)	IVT	0.36	Eastern Canada, Western and southwest U.S.	0.20–0.30	7	Mexico
	IWV	0.45+	North of Quebec and Newfoundland, west of the U.S.	0.18–0.28	4	None (max 4)
Summer (JJA)	IVT	0.38	Eastern Canada,	0.14–0.24	5	British Columbia, North of Canada
	IWV	0.45+	North of Quebec and Newfoundland, Northwest Territories	0.12–0.22	4	None (max 4)
Autumn (SON)	IVT	0.30	Newfoundland and Labrador	0.10–0.20	4	None (max 4)
	IWV	0.45+	North of Canada	0.15–0.25	4	None (max 4)
Winter (DJF)	IVT	0.45+	East of North America, Greenland, Mexico	0.30–0.40	4	None (max 4)
	IWV	0.45+	Canada, Greenland, Mexico	0.35–0.45	6	South of Alaska, California, Mexico, South of Greenland

**Fig. 8:**
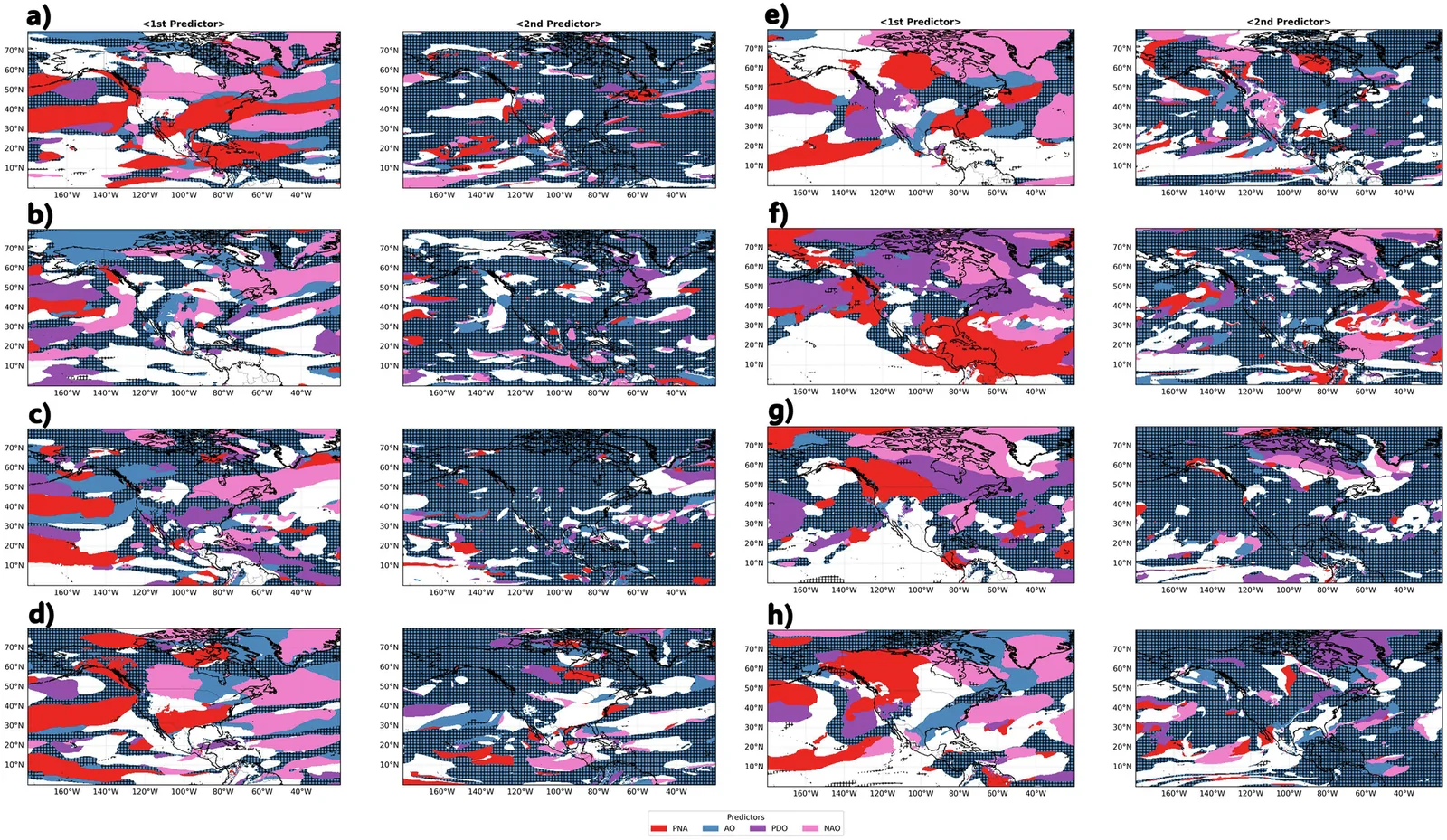
Influential teleconnection predictors for regional AR variability. The most influential teleconnection predictors of the modified IVT and IWV indices are identified based on stepwise multiple linear regression analysis across the four seasons. **a**–**d** Display the first and second most dominant large-scale climate predictors for IVT during spring, summer, autumn, and winter, respectively, while (**e**–**h**) illustrate the corresponding predictors for IWV during the same seasons. These maps highlight the relative influence of the Pacific–North American pattern (PNA; red), Arctic Oscillation (AO; blue), Pacific Decadal Oscillation (PDO; purple), and North Atlantic Oscillation (NAO; pink) on atmospheric river variability. Black stippling (dots) indicates regions where predictor dominance is statistically significant at the 95% confidence level. Collectively, these results reveal distinct seasonal contrasts and spatial dependencies in how major teleconnections modulate atmospheric river intensity and moisture transport across North America.

Stepwise regression analysis identified AO, PNA, PDO, and NAO as the dominant large-scale teleconnection modes governing AR activity across the study area, each exerting spatially distinct effects. For instance, in contrast to ENSO and Niño 3.4, which exhibited comparable effects in the southern domain, the NAO showed a more substantial effect over eastern NA, while the PNA emerged as the dominant driver in the west. However, the influence of ENSO and Niño 3.4 decreased at mid-latitudes, where the PNA and PDO proved to be the primary drivers. Furthermore, referring to the second predictor maps, the most important predictor reveals that PDO exerts dominant control over IWV in northern North America during autumn, winter, and summer, with extensive spatial coverage in high-latitude regions (Greenland, northern Canada) across all three seasons (Fig. 8).

These results reveal that, despite broadly similar overall correlations with AR frequency, the regional expression of these modes differs markedly, a pattern consistent with that of Dong et al. (2025). According to their analysis, the PNA–IVT relationship reflects two anticyclonic powers: a robust center along the western coast and a weaker one over the Gulf of Mexico. In western NA, the intensified western anticyclone enhances northward advection of cold, dry air, thereby inhibiting eastward penetration of ARs into the continental interior^79^. Conversely, strengthened anticyclonic flow near the eastern seaboard^80^ favors convergence between warm, moist air from the Gulf of Mexico and Atlantic and cold, dry air from higher latitudes, intensifying AR formation and associated precipitation along the eastern coast. This low-level convergence zone, often accompanied by adjustments in the position and strength of the mid-latitude jet stream^81^. Furthermore, Dong et al. (2025) documented those anticyclonic anomalies, linked to the declining PNA index, have amplified over the past four decades, reinforcing the pronounced east–west asymmetry in AR dynamics.

## Discussion

This study investigated four decades of AR activity across North America, elucidating the dynamic and thermodynamic mechanisms modulating their frequency and intensity. By integrating upper-tropospheric thermodynamic constraints into the traditionally established IVT and IWV frameworks, we developed modified indices that were rigorously validated against independent radiosonde profiles and SSMIS retrievals, confirming superior performance in capturing the vertical structure of the atmosphere. Our findings demonstrate that these modifications effectively mitigate systematic biases in AR diagnosis, enabling a more reliable identification of narrow filaments and a more precise quantification of integrated moisture. Specifically, the modified indices improved AR structural representation by up to 8.7% and increased the detection efficiency for narrow ARs from 79% to 91%. Beyond technical precision, these advances offer a robust framework for improving hydro-meteorological forecasting and flood-risk mitigation. Seasonal trend analysis (1984–2024) revealed a pronounced spatial heterogeneity in AR evolution. While positive trends dominate most seasons, a significant east–west asymmetry emerged, with the most substantial intensifications concentrated over eastern North America. This continental-scale asymmetry reinforces recent findings of localized increases in the Eastern US^82^, providing a broader geographic context for the observed intensifications. This regional divergence is characterized by decadal slopes of 0.1–0.9 mm decade⁻¹ for IWV and 0.1–0.9 kg m⁻¹ s⁻¹ decade⁻¹ for IVT. The high degree of synchronicity between IWV and IVT trends confirms that AR intensification is driven by a coupled strengthening of both thermodynamic and dynamic processes. Furthermore, our stepwise regression analysis identified the PNA, AO, PDO, and NAO as the primary dynamical drivers of AR variability. These results expand upon sub-seasonal frameworks focusing on the West Pacific modulation of coastal ARs^78^ and align with the evolving phase shifts and jet core drifts identified in Northern Hemisphere stationary waves^83^. The NAO exerts a dominant steering influence over eastern NA, while the PNA dictates AR landfalls in the west, with both indices shifting dominance according to seasonal transitions. Crucially, the fundamental novelty of this climate-driver analysis lies in the significantly enhanced physical fidelity of the diagnostic indices. Unlike conventional formulations that focus primarily on bulk moisture transport, our ‘sharpened’ indices incorporate upper-level thermodynamics, including stratospheric–tropospheric interactions and PV intrusions. This refined framework enables a more physically robust representation of how large-scale circulation patterns modulate regional AR activity and condensation efficiency—variability that remains largely uncaptured by standard IVT/IWV metrics. While establishing definitive causal linkages remains complex due to the nonlinear nature of global circulation, the teleconnection insights and tropopause-aware indices presented here provide a vital physical foundation for advancing the predictability of extreme precipitation and flood hazards across North America.

## Methods

### Data and study period

In this study, high-resolution ERA5 reanalysis data from the European Centre for Medium-Range Weather Forecasts (ECMWF) were analyzed to characterize the structure and frequency of ARs over NA and adjacent oceanic domains from 1984 through 2024. ERA5 supplies hourly output on a 0.25° × 0.25° grid across pressure levels from 1000 hPa to 10 hPa. The variables extracted included specific humidity (*q*), zonal and meridional winds (*u*, *v*), temperature (*T*), geopotential height (hgt), and pressure (*P*). To anchor the reanalysis against real atmospheric soundings, radiosonde observations from a network of stations across NA, with special attention to Hawaii, strategically positioned along the primary corridor for narrow Pacific ARs, were incorporated. These vertical profiles of pressure, temperature, humidity, and wind at different atmospheric levels were drawn from the Integrated Global Radiosonde Archive (IGRA). Additionally, the spatial extent and intensity of the moisture plumes were validated using Special Sensor Microwave Imager/Sounder (SSMIS) satellite imagery. To reinforce these findings, we conducted a dynamic analysis of the equivalent potential temperature (EQPT) and the dynamical coupling between stratospheric potential vorticity (PV) and low-level specific humidity. These integrated physical lines of evidence confirm a high degree of consistency between the proposed modified diagnostic indices and the observed AR structures.

### Lagrangian-based 4D collocation of radiosonde and ERA5 data

**Fig. 9:**
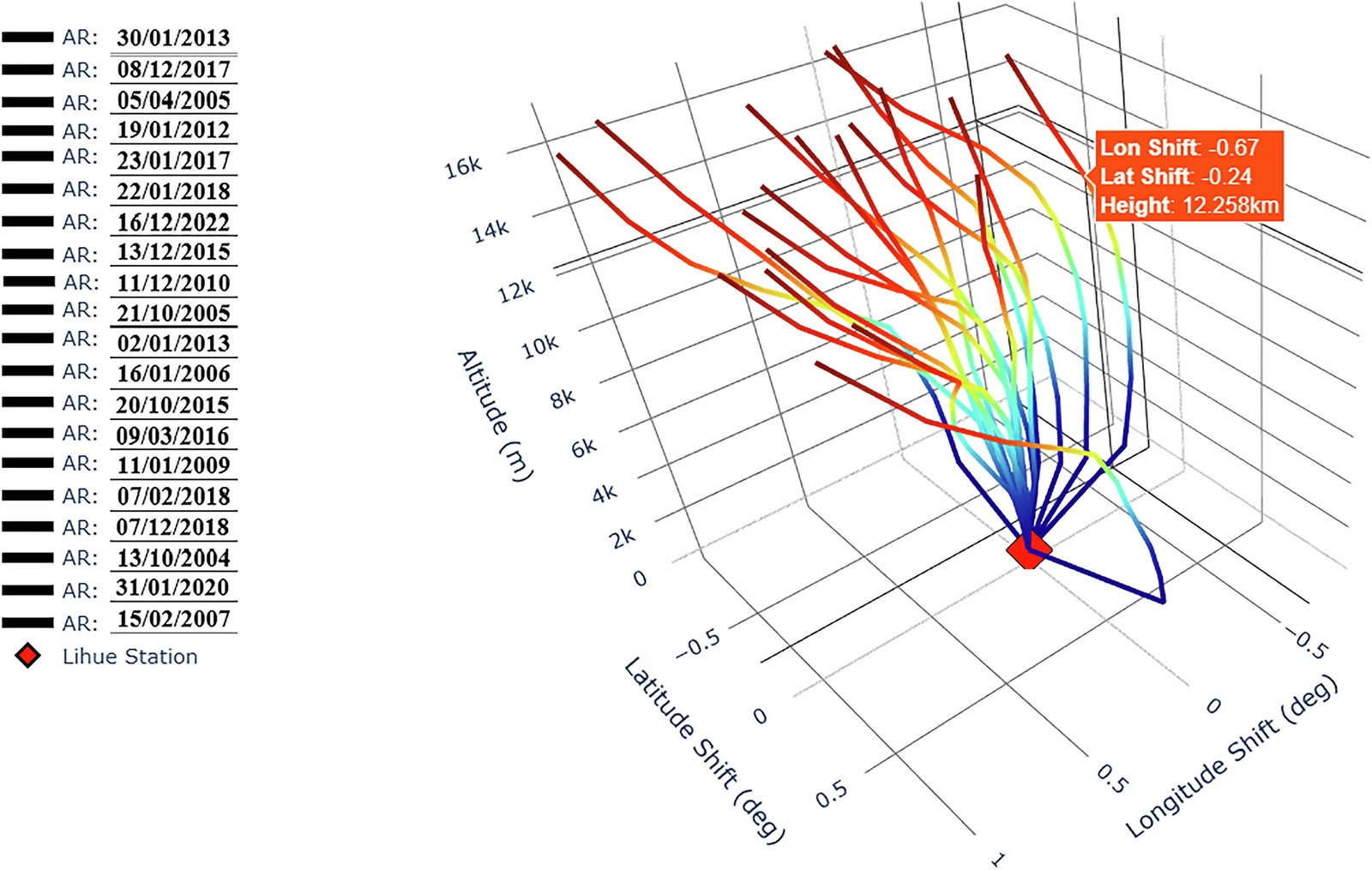
4D Lagrangian reconstruction of radiosonde trajectories. A 4D Lagrangian trajectory reconstruction was performed for 20 selected atmospheric river events at Lihue Station, Hawaii, to ensure spatiotemporal consistency between in-situ observations and reanalysis data. The paths illustrate the horizontal drift of the radiosonde balloons relative to the launch site, indicated by the red square, as a function of altitude and time. Color transitions along the trajectories represent the change in altitude from the surface up to 16 km, while the axes show the specific shifts in longitude and latitude. This methodology facilitates a rigorous 4D collocation, ensuring that ERA5 grid variables are extracted at the precise location and time-step of the balloon’s presence during high-intensity moisture-transport events.

In this study, a 4D Lagrangian trajectory reconstruction (Fig. 9) was performed to ensure the highest degree of spatiotemporal consistency between in-situ observations and model data. For this purpose, 20 specific radiosonde soundings were randomly selected (Table 1) from historical AR events passing over the Lihue Airport Station, Hawaii.

The primary objective of this reconstruction was to facilitate a rigorous comparison between standard ERA5 outputs and the radiosonde observations. The trajectory for each of the 20 cases was calculated by integrating the observed zonal and meridional wind (u, v) components at each pressure level (*P*) to determine the exact geographical coordinates $\left({\rm{\lambda }},{\rm{\phi }}\right)$ and the corresponding time (*t*) of the sensor as it drifted away from the launch site:

1 $${{\rm{\lambda }}}_{i+1}={{\rm{\lambda }}}_{i}+{\int }_{{t}_{i}}^{{t}_{i+1}}\frac{u\left(t\right)}{R\cos \left({\rm{\phi }}\right)}\,{dt}$$

2 $${{\rm{\phi }}}_{i+1}={{\rm{\phi }}}_{i}+{\int }_{{t}_{i}}^{{t}_{i+1}}\frac{v\left(t\right)}{R}\,{dt}$$

Where R is the Earth’s radius, $\left(\lambda ,\phi \right)$ represent longitude and latitude, and dt denotes the temporal resolution (time step) of the radiosonde sampling. This methodology allows for a 4D spatiotemporal collocation, ensuring that ERA5 grid variables are extracted at the precise location and time-step of the balloon’s presence. By considering the horizontal drift, this method reduces errors and creates a strong system for checking reanalysis products against high-intensity moisture transport. This approach is consistent with previous findings which indicate that the agreement between profiling platforms can be significantly improved by considering the advection of air parcels by the wind^84^.

### ERA5-based formulations of IVT and IWV

The ERA5 dataset was used to calculate two fundamental vapor transport indices:

Integrated Vapor Transport (IVT): representing the meridional and horizontal flux of water vapor:

3 $$\mathrm{IVT}={{\rm{g}}}^{-1}\sqrt{{\left({\int }_{{{\rm{p}}}_{\mathrm{surface}}}^{{\rm{p}}}\mathrm{qU}\,\mathrm{dp}\right)}^{2}+{\left({\int }_{{{\rm{p}}}_{\mathrm{surface}}}^{{\rm{p}}}\mathrm{qV}\,\mathrm{dp}\right)}^{2}}$$

Integrated Water Vapor (IWV): expressing the total atmospheric column of water vapor:

4 $${\rm{IWV}}={{\rm{g}}}^{-1}{\int }_{{{\rm{p}}}_{{\rm{surface}}}}^{{\rm{p}}}{\rm{q\; dp}}$$

Where p is specified pressure level, q is the specific humidity (g·kg⁻¹), U and V are the zonal and meridional components of wind (m·s⁻¹), and g is the acceleration due to gravity (9.81 m·s⁻²).

These parameters were computed across all grid points and time steps to detect ARs characterized by enhanced meridional moisture transport.

### Thermodynamic adjustment and IWV and IVT modification

The standard IVT and IWV formulations often underestimate localized, vertically unstable AR cores due to their limited sensitivity to tropospheric temperature gradients. To address this limitation, a thermodynamic correction was introduced through the incorporation of a vertical temperature gradient term:

5 $$\nabla {{\rm{T}}}_{\mathrm{trop}}=\frac{\mathrm{dT}}{{\rm{d}}(\mathrm{hgt})}=\frac{{\rm{T}}({{\rm{p}}}_{2})-{\rm{T}}({{\rm{p}}}_{1})}{\mathrm{hgt}({{\rm{p}}}_{2})-\mathrm{hgt}({{\rm{p}}}_{1})}\times 1000$$

where $T\left({p}_{1}\right)$ and $T({p}_{2})$ denote the mean temperatures at 150 hPa and 300 hPa, respectively, and ${hgt}$ refers to the geopotential height (m). This parameter ($\nabla {T}_{{trop}}$) quantifies vertical instability and energy exchange within the troposphere, which are key mechanisms driving AR intensity and condensation efficiency.

### Thermodynamic weighting function

To integrate the temperature gradient effect into the transport equations, a weighting function (*W*) was designed as follows:

6 $${\rm{W}}=1+{\rm{a}}\cdot \frac{\nabla {{\rm{T}}}_{\mathrm{trop}}}{\nabla {{\rm{T}}}_{\mathrm{ref}}}$$

Where *a* = 0.5 is an empirical coefficient derived from radiosonde calibration, and ($\nabla {T}_{\mathrm{ref}}$) represents the long-term climatological mean gradient calculated over 1984–2024. The value of a was optimized to minimize systematic discrepancies between reanalysis indices and in-situ radiosonde profiles, ensuring the sensitivity of the modification is physically consistent with observed atmospheric structures. Crucially, while a serves as a static calibration constant, the resulting weighting factor W is inherently dynamic. Since *W* is computed using the localized, hourly vertical temperature gradient at each grid point, the function automatically accounts for spatiotemporal variability across various latitudes and seasons. The term *W* dynamically scales moisture-transport intensity based on vertical instability, amplifying IVT and IWV in regions with strong temperature gradients and latent heat release.

### Modified IVT and IWV formulations

The final modified versions of the indices were expressed as:

7 $$\mathrm{IV}{{\rm{T}}}_{\mathrm{modified}}=\mathrm{IVT}\cdot W\left(\nabla {T}_{\mathrm{trop}}\right)$$

8 $$\mathrm{IW}{{\rm{V}}}_{\mathrm{modified}}=\mathrm{IWV}\cdot W(\nabla {T}_{\mathrm{trop}})$$

These enhanced forms provide better physical representation of ARs by linking thermodynamic processes (via temperature gradients) with dynamic moisture transport, enabling improved detection of narrow and intense AR filaments that standard methods often miss.

### Radiosonde and satellite validation

To evaluate the accuracy of the modified indices, comparisons were made with radiosonde-derived observations at 850 hPa, which referring to previous studies, this pressure level represents the AR moisture core. The observed IVT and IWV were calculated as:

9 $${\rm{IV}}{{\rm{T}}}_{{\rm{obs}}}={{\rm{g}}}^{-1}\sqrt{{\left({\int }_{{{\rm{p}}}_{{\rm{surface}}}}^{{\rm{p}}}{\rm{qU}}\,{\rm{dp}}\right)}^{2}+{\left({\int }_{{{\rm{p}}}_{{\rm{surface}}}}^{{\rm{p}}}{\rm{qV}}\,{\rm{dp}}\right)}^{2}}$$

10 $${\mathrm{IWV}}_{{\rm{obs}}}=\frac{1}{g}\mathop{\sum }\limits_{i}{q}_{i}\,\Delta {p}_{i}$$

Twenty representative AR events over Hawaii were selected to assess both temporal and spatial consistency between the modified IWV and IVT, standard IWV and IVT outputs, and the radiosonde dataset. Moreover, SSMIS satellite imagery provided further qualitative confirmation of AR boundaries and moisture intensity, verifying that the modified indices better captured the observed vapor plume geometry.

### Statistical evaluation metrics

To quantify performance, the following statistical measures were applied between the diagnostic indices and observed parameters:

11 $$\begin{array}{l}\mathrm{Root}\,\mathrm{Mean}\,\mathrm{Square}\,\mathrm{Error}\\ \mathrm{RMSE}=\sqrt{\frac{1}{{\rm{n}}}\Sigma {({{\rm{x}}}_{{\rm{i}}}-{{\rm{y}}}_{{\rm{i}}})}^{2}}\end{array}$$

12 $$\begin{array}{l}\mathrm{Mean}\,\mathrm{Absolute}\,\mathrm{Error}\\ \mathrm{MAE}=\frac{1}{{\rm{n}}}\sum {\rm{| }}{{\rm{x}}}_{{\rm{i}}}-{{\rm{y}}}_{{\rm{i}}}{\rm{| }}\end{array}$$

13 $${\rm{Bias}}=\frac{1}{{\rm{n}}}\Sigma ({{\rm{x}}}_{{\rm{i}}}-{{\rm{y}}}_{{\rm{i}}})$$

14 $$\begin{array}{c}\mathrm{Pearson}\,\mathrm{Correlation}\mathrm{Coefficient}\\ {\rm{r}}=\frac{\Sigma ({{\rm{x}}}_{{\rm{i}}}-\bar{{\rm{x}}})({{\rm{y}}}_{{\rm{i}}}-\bar{{\rm{y}}})}{\sqrt{\Sigma {({{\rm{x}}}_{{\rm{i}}}-\bar{{\rm{x}}})}^{2}\Sigma {({{\rm{y}}}_{{\rm{i}}}-\bar{{\rm{y}}})}^{2}}}\end{array}$$

15 $$\begin{array}{l}\mathrm{Relative}\,\mathrm{Improvement}\\ \mathrm{RI}( \% )=\frac{{\mathrm{RMSE}}_{(\mathrm{IWV}\& \mathrm{IVT})\mathrm{std}}-{\mathrm{RMSE}}_{(\mathrm{IWV}\& \mathrm{IVT})\mathrm{modified}}}{{\mathrm{RMSE}}_{(\mathrm{IWV}\& \mathrm{IVT})\mathrm{std}}}\times 100\end{array}$$

The metrics were calculated for both IVT and IWV at daily intervals during AR occurrences.

### Long-term atmospheric river analysis

Following validation, the modified diagnostic indices were applied to the whole 1984–2024 ERA5 dataset to construct a 40-year climatology of AR frequency, intensity, and distribution over NA. Finally, substantial seasonal variability and relationships between AR intensity and 12 major teleconnection indices, including AO, NAO, PNA, PDO, MJO, CENSO, MEIv2, and ENSO phases (Niño 1 + 2, Niño 3, Niño 3.4, Niño 4, and Oceanic Niño indices ONI), were examined to assess the sensitivity of AR activity to large-scale atmospheric circulation. Finally, to determine the most influential teleconnection patterns modulating ARs and to evaluate their regional differences, stepwise multiple linear regression (MLR) was applied.

## Supplementary information

Supplementary information

## Data Availability

The datasets used and/or analyzed during the current study are available from the corresponding author upon reasonable request. The underlying code for this study is not publicly available but may be made available to qualified researchers at a reasonable request from the corresponding author.

## Code availability

The underlying code for this study is not publicly available but may be made available to qualified researchers at a reasonable request from the corresponding author.
