# Supplementary material for: Tropopause-aware IVT and IWV sharpen atmospheric river detection over North America
Source: NPJ Clim Atmos Sci. 2026 Jun 4;9(1):180. doi: 10.1038/s41612-026-01449-x (PMC13553291; doi:10.1038/s41612-026-01449-x)
Supplement: Supplementary file 1 [file 41612_2026_1449_MOESM1_ESM.pdf]

Supplementary information for

Tropopause-aware IVT and IWV sharpen atmospheric river detection over North America

Saadoun Salimi[1]*, Taha B.M.J. Ouarda[1]

1. Canada Research Chair in Statistical Hydro-Climatology, Institut National de la Recherche Scientifique, Centre Eau Terre Environnement, INRS-ETE, 490 de la Couronne, Québec, QC, G1K 9A9, Canada

*Corresponding author: 490, Couronne street, Québec, QC, G1K 9A9, Canada.
E-mail address: Saadoun.Salimi@inrs.ca (S. Salimi).

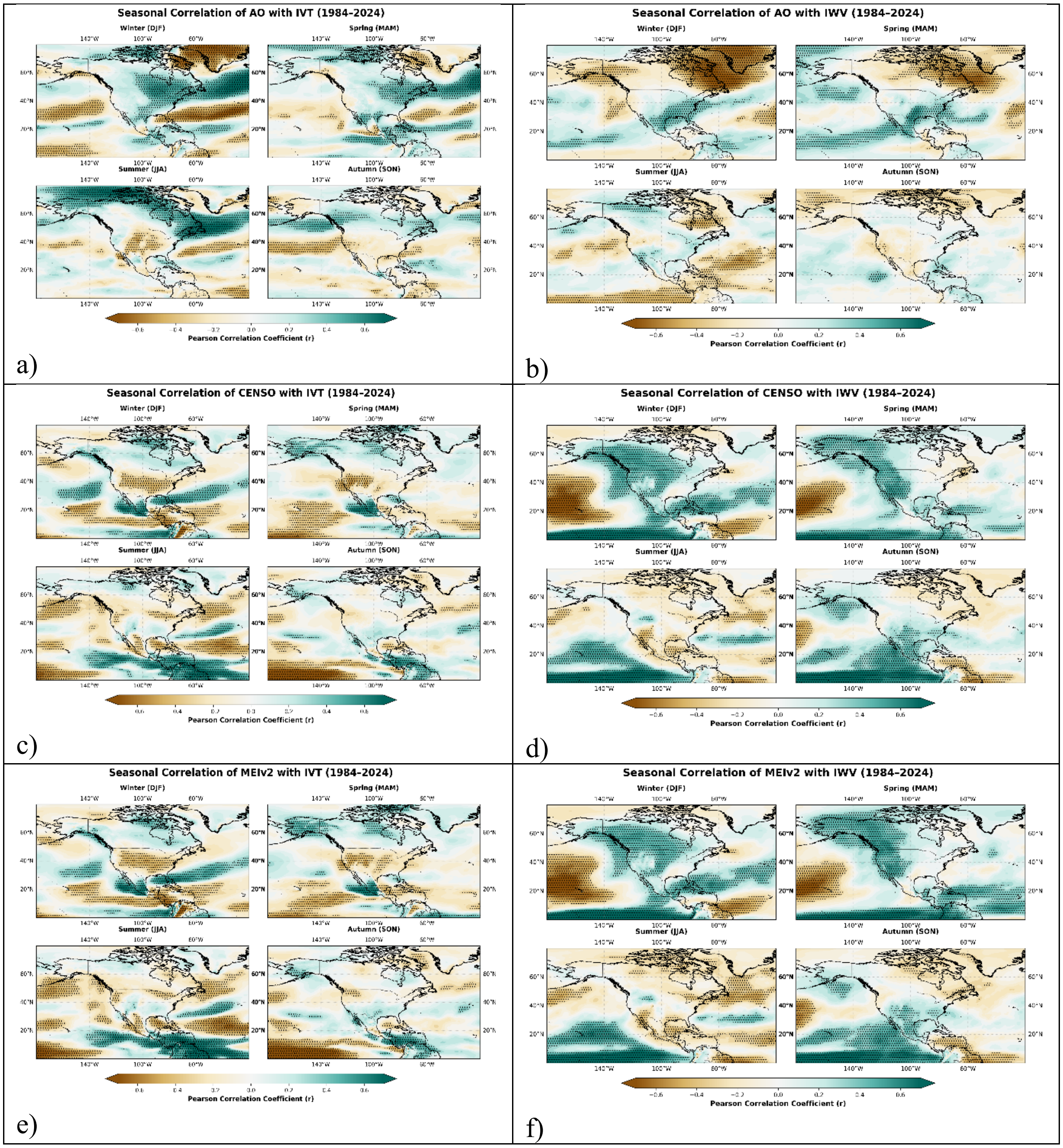
Seasonal Correlation of AO with IVT (1984–2024)
Winter (DJF)
Spring (MAM)
Summer (JJA)
Autumn (SON)
Pearson Correlation Coefficient (r)
a)
Seasonal Correlation of AO with IWV (1984–2024)
Winter (DJF)
Spring (MAM)
Summer (JJA)
Autumn (SON)
Pearson Correlation Coefficient (r)
b)
Seasonal Correlation of CENSO with IVT (1984–2024)
Winter (DJF)
Spring (MAM)
Summer (JJA)
Autumn (SON)
Pearson Correlation Coefficient (r)
c)
Seasonal Correlation of CENSO with IWV (1984–2024)
Winter (DJF)
Spring (MAM)
Summer (JJA)
Autumn (SON)
Pearson Correlation Coefficient (r)
d)
Seasonal Correlation of MEIv2 with IVT (1984–2024)
Winter (DJF)
Spring (MAM)
Summer (JJA)
Autumn (SON)
Pearson Correlation Coefficient (r)
e)
Seasonal Correlation of MEIv2 with IWV (1984–2024)
Winter (DJF)
Spring (MAM)
Summer (JJA)
Autumn (SON)
Pearson Correlation Coefficient (r)
f)

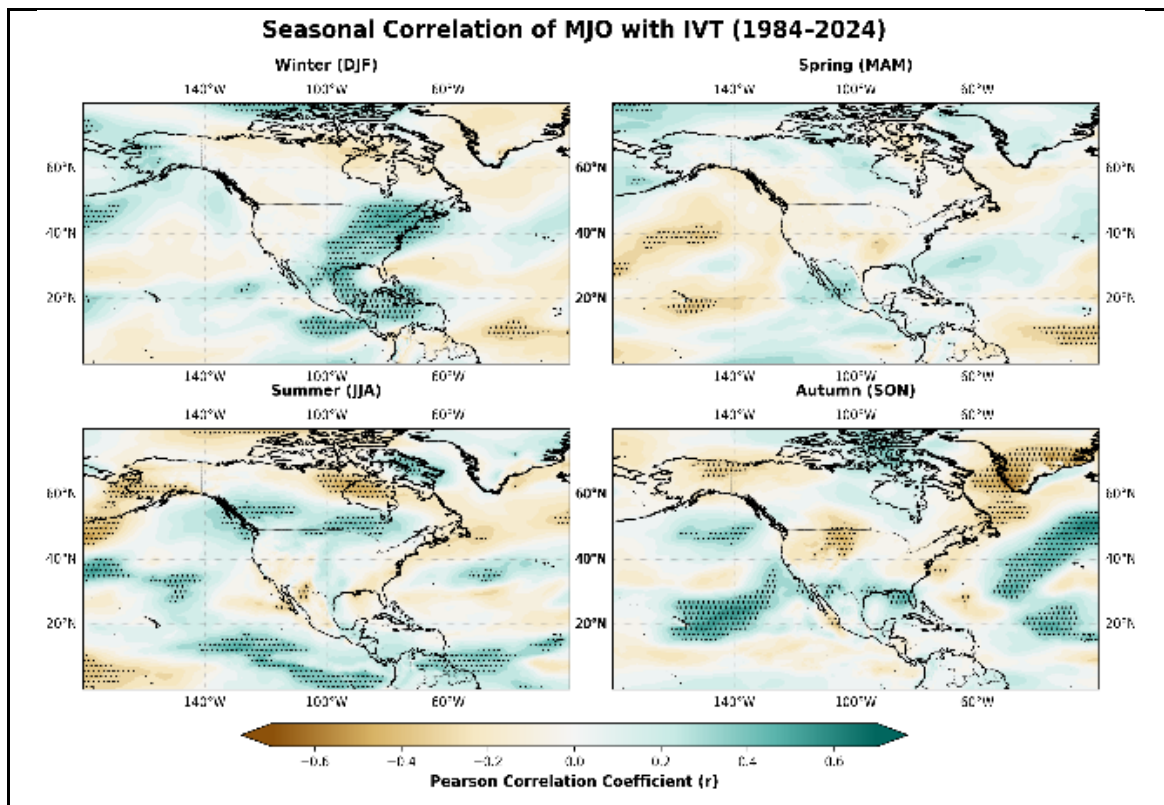
Seasonal Correlation of MJO with IVT (1984–2024)
Winter (DJF)
Spring (MAM)
Summer (JJA)
Autumn (SON)
Pearson Correlation Coefficient (r)

g)

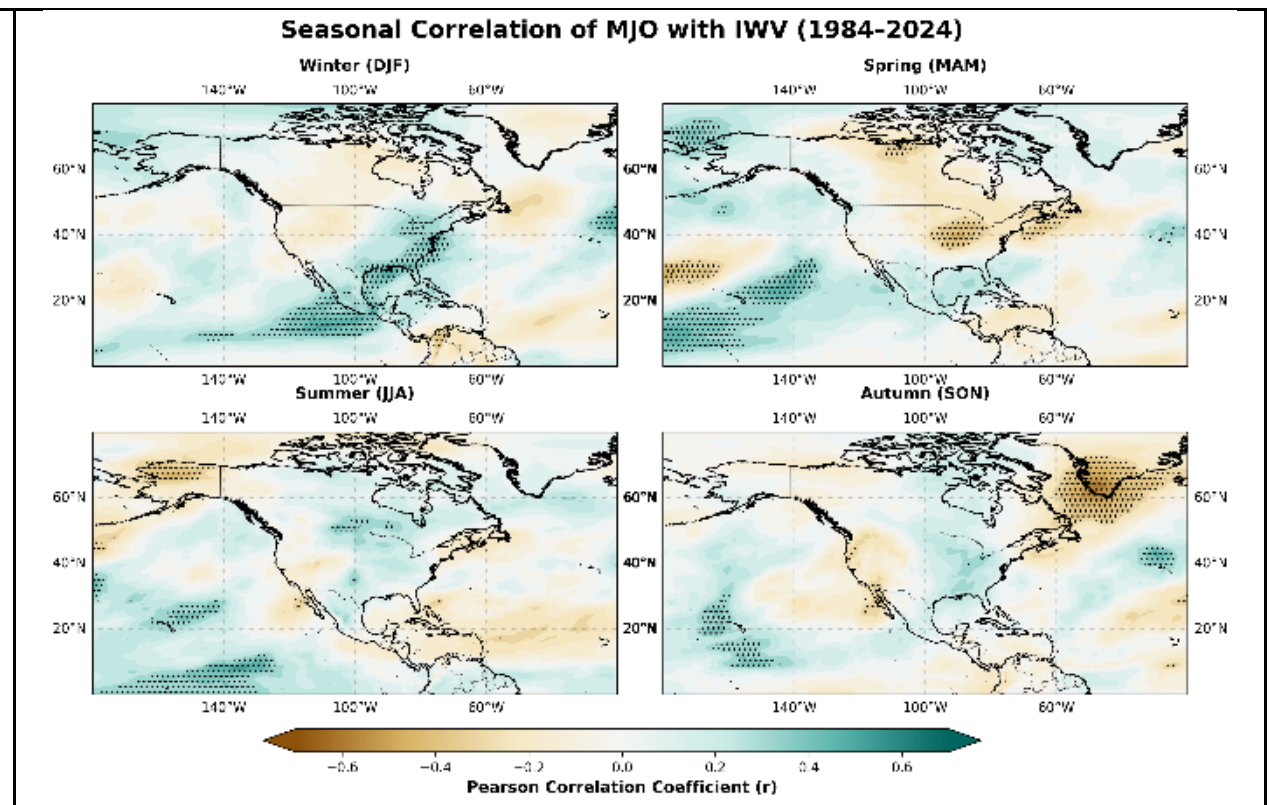
Seasonal Correlation of MJO with IWV (1984–2024)
Winter (DJF)
Spring (MAM)
Summer (JJA)
Autumn (SON)
Pearson Correlation Coefficient (r)

h)

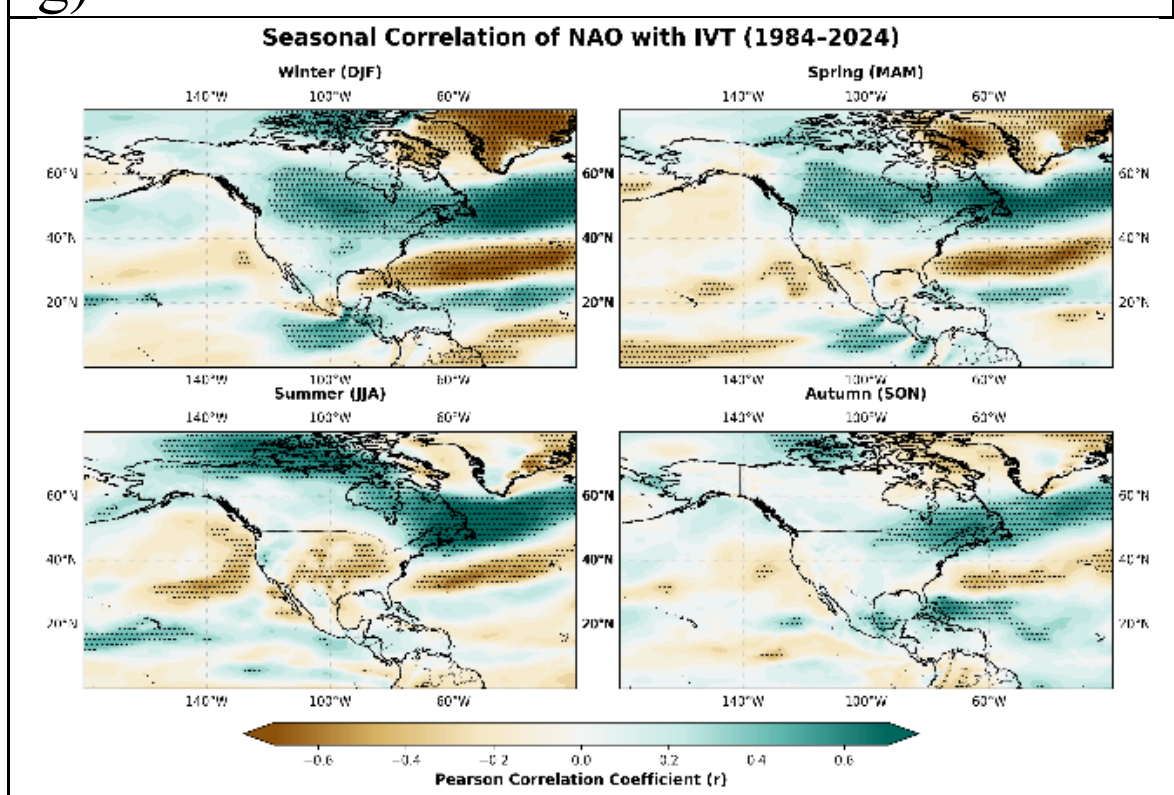
Seasonal Correlation of NAO with IVT (1984–2024)
Winter (DJF)
Spring (MAM)
Summer (JJA)
Autumn (SON)
Pearson Correlation Coefficient (r)

i)

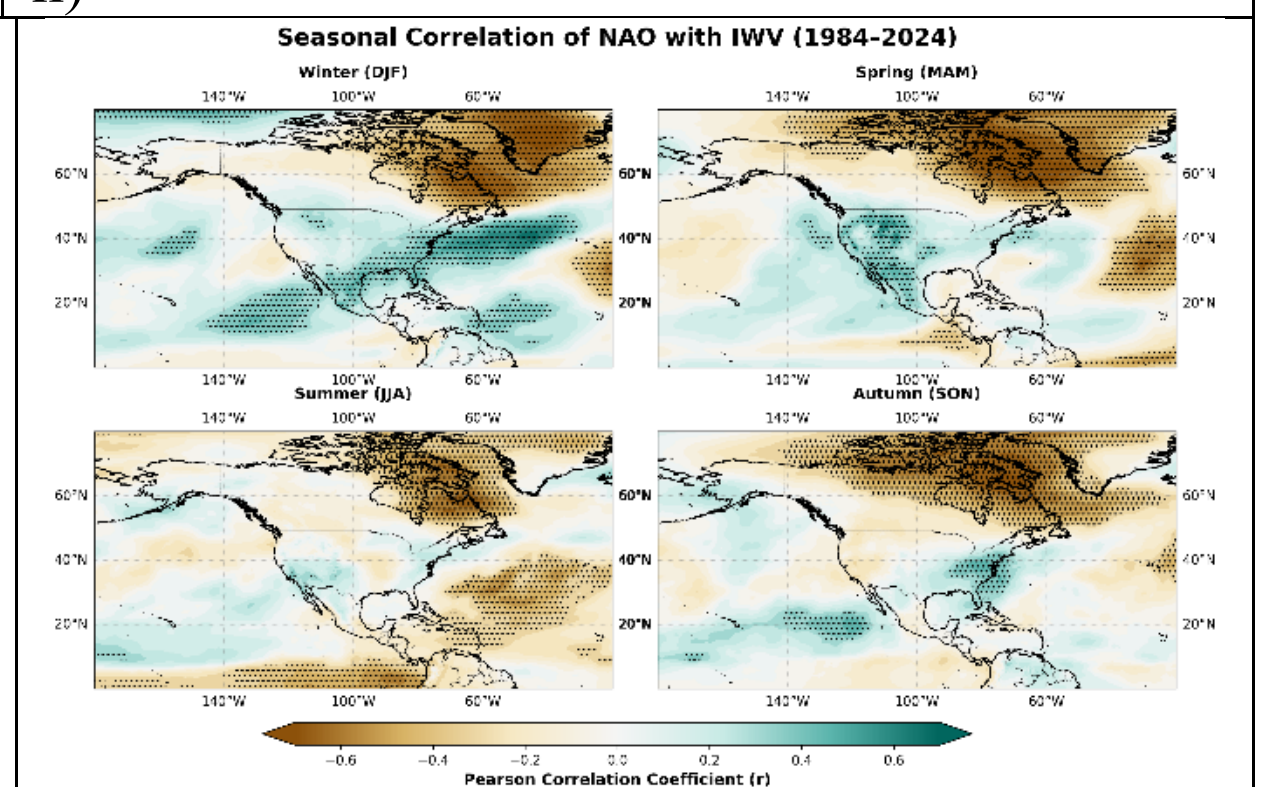
Seasonal Correlation of NAO with IWV (1984–2024)
Winter (DJF)
Spring (MAM)
Summer (JJA)
Autumn (SON)
Pearson Correlation Coefficient (r)

j)

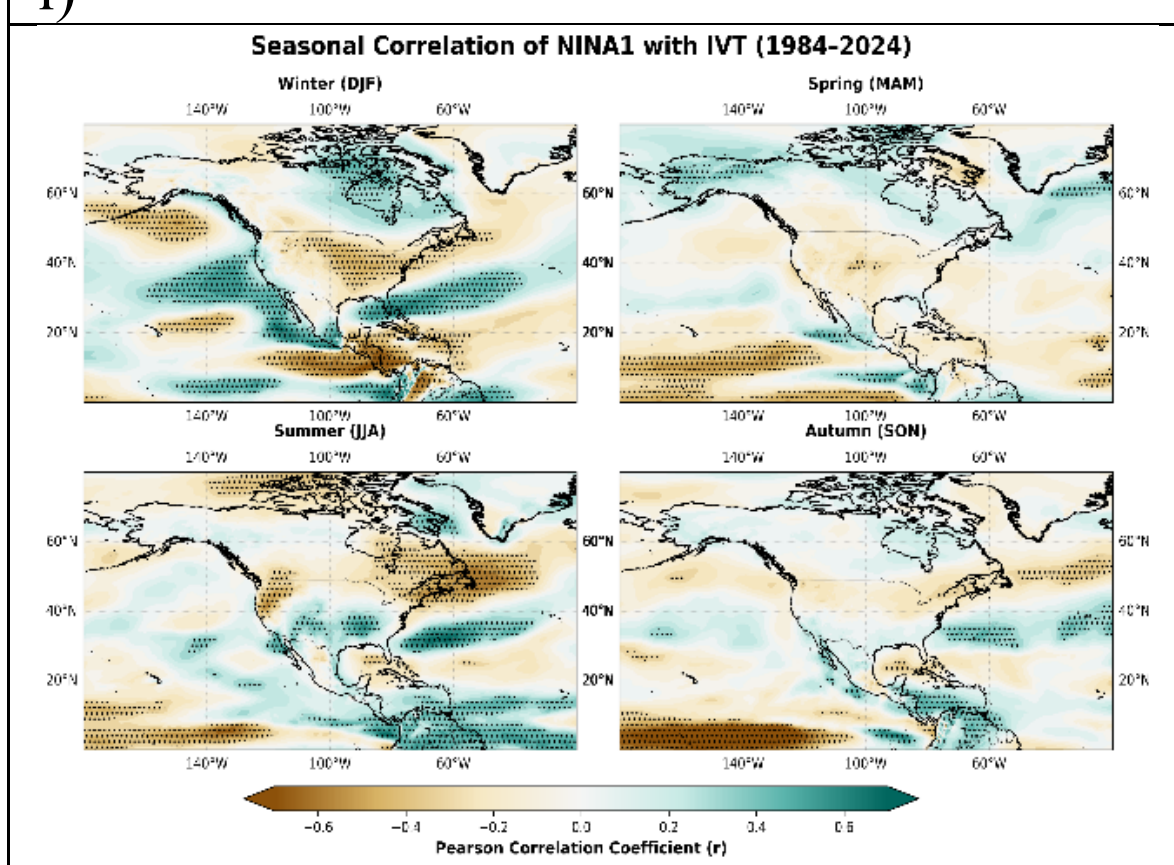
Seasonal Correlation of NINA1 with IVT (1984–2024)
Winter (DJF)
Spring (MAM)
Summer (JJA)
Autumn (SON)
Pearson Correlation Coefficient (r)

k)

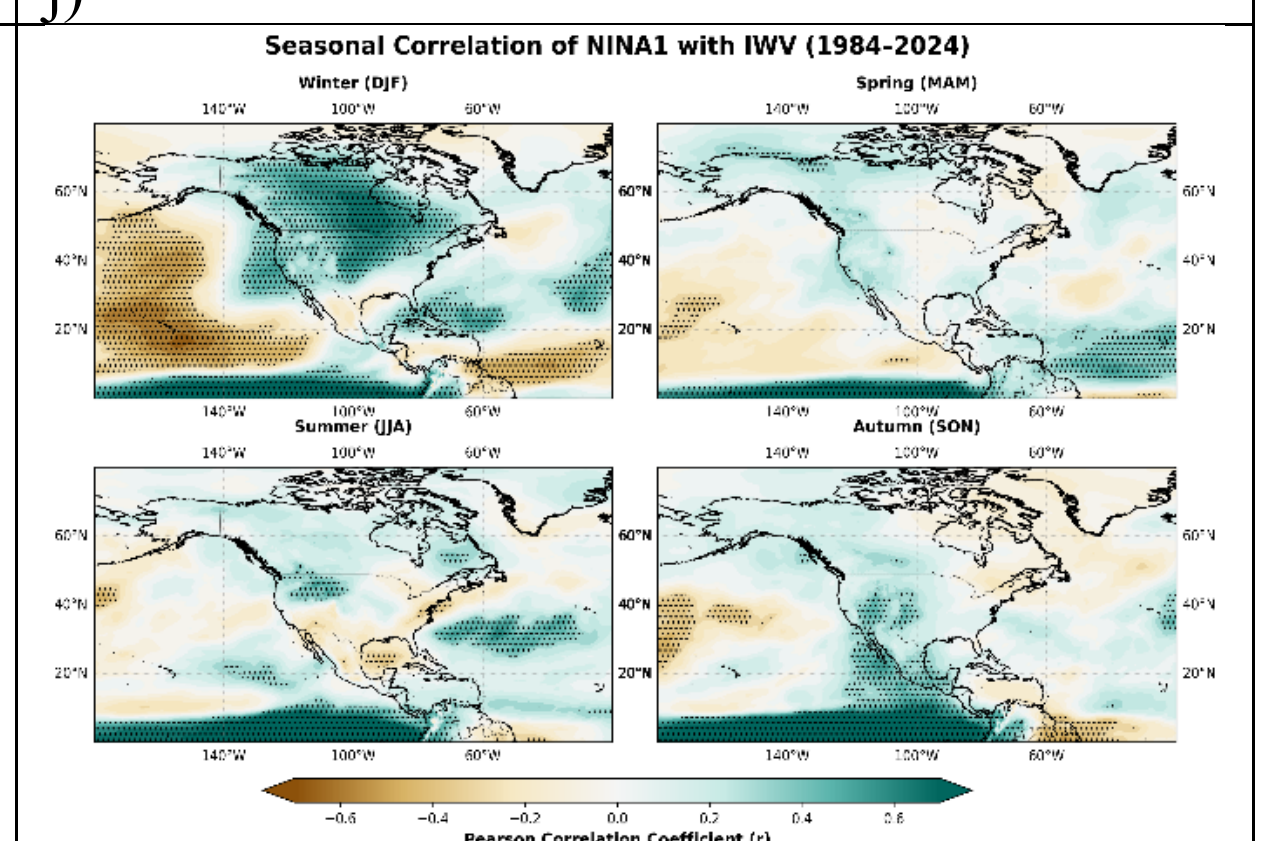
Seasonal Correlation of NINA1 with IWV (1984–2024)
Winter (DJF)
Spring (MAM)
Summer (JJA)
Autumn (SON)
Pearson Correlation Coefficient (r)

l)

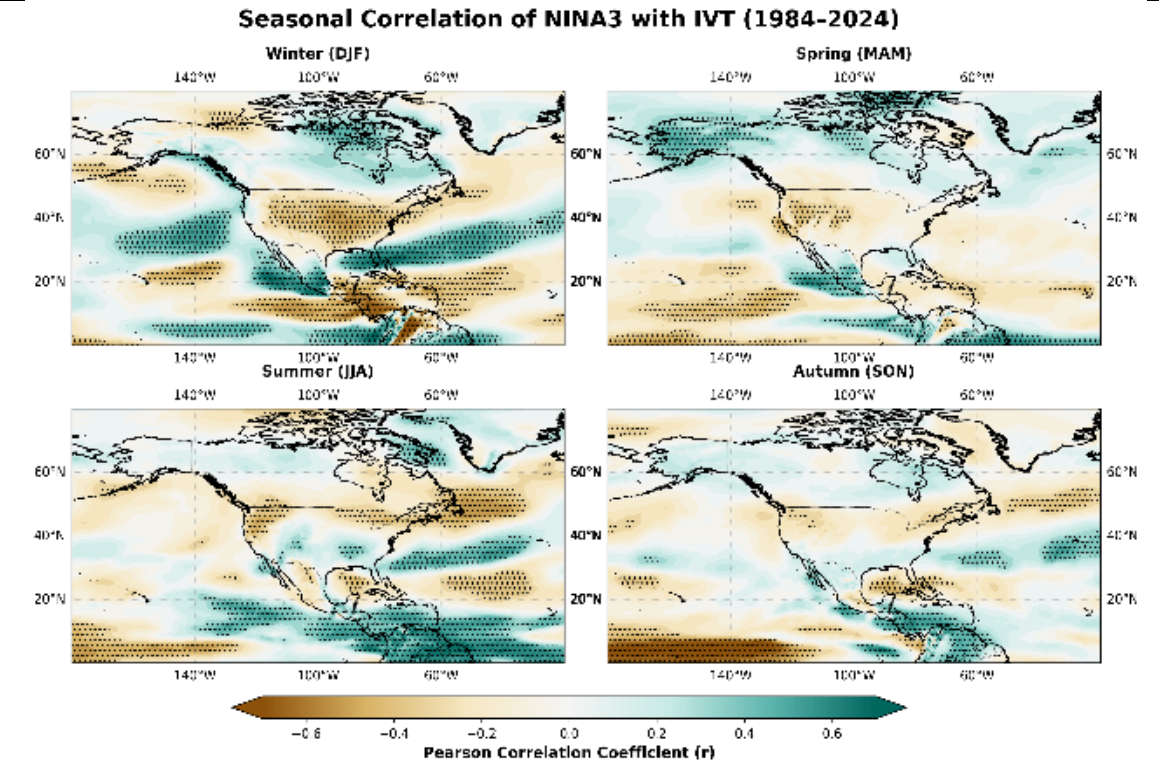
Seasonal Correlation of NINA3 with IVT (1984–2024)
Winter (DJF)
Spring (MAM)
Summer (JJA)
Autumn (SON)
Pearson Correlation Coefficient (r)

m)

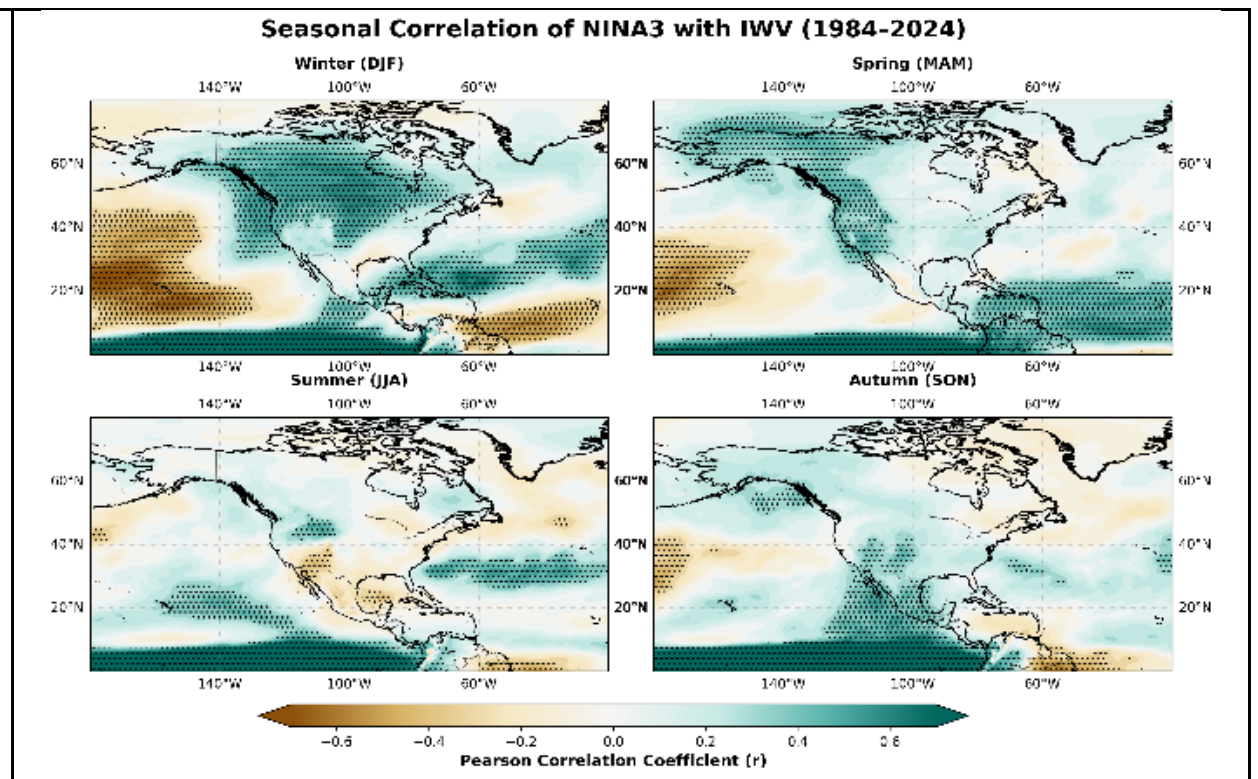
Seasonal Correlation of NINA3 with IWV (1984–2024)
Winter (DJF)
Spring (MAM)
Summer (JJA)
Autumn (SON)
Pearson Correlation Coefficient (r)

n)

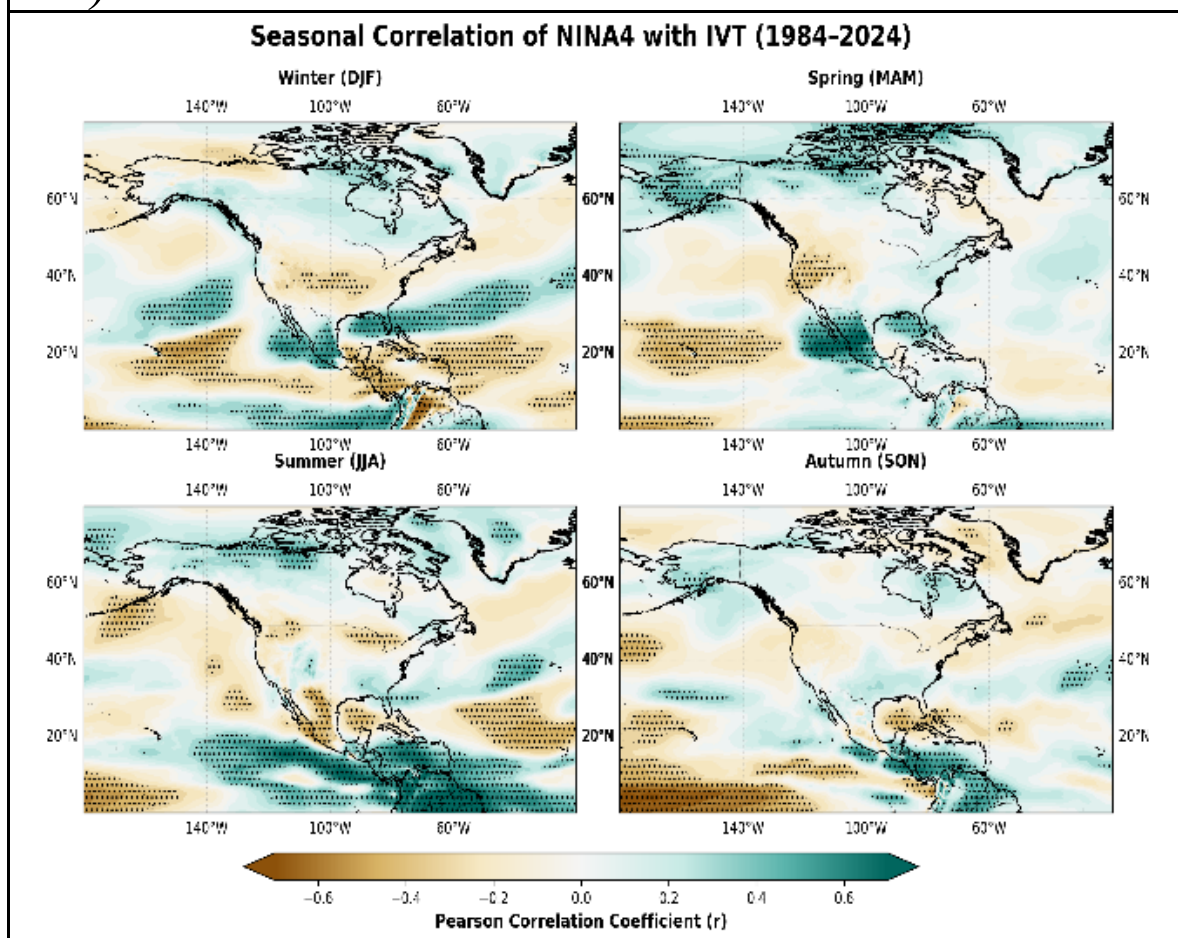
Seasonal Correlation of NINA4 with IVT (1984–2024)
Winter (DJF)
Spring (MAM)
Summer (JJA)
Autumn (SON)
Pearson Correlation Coefficient (r)

o)

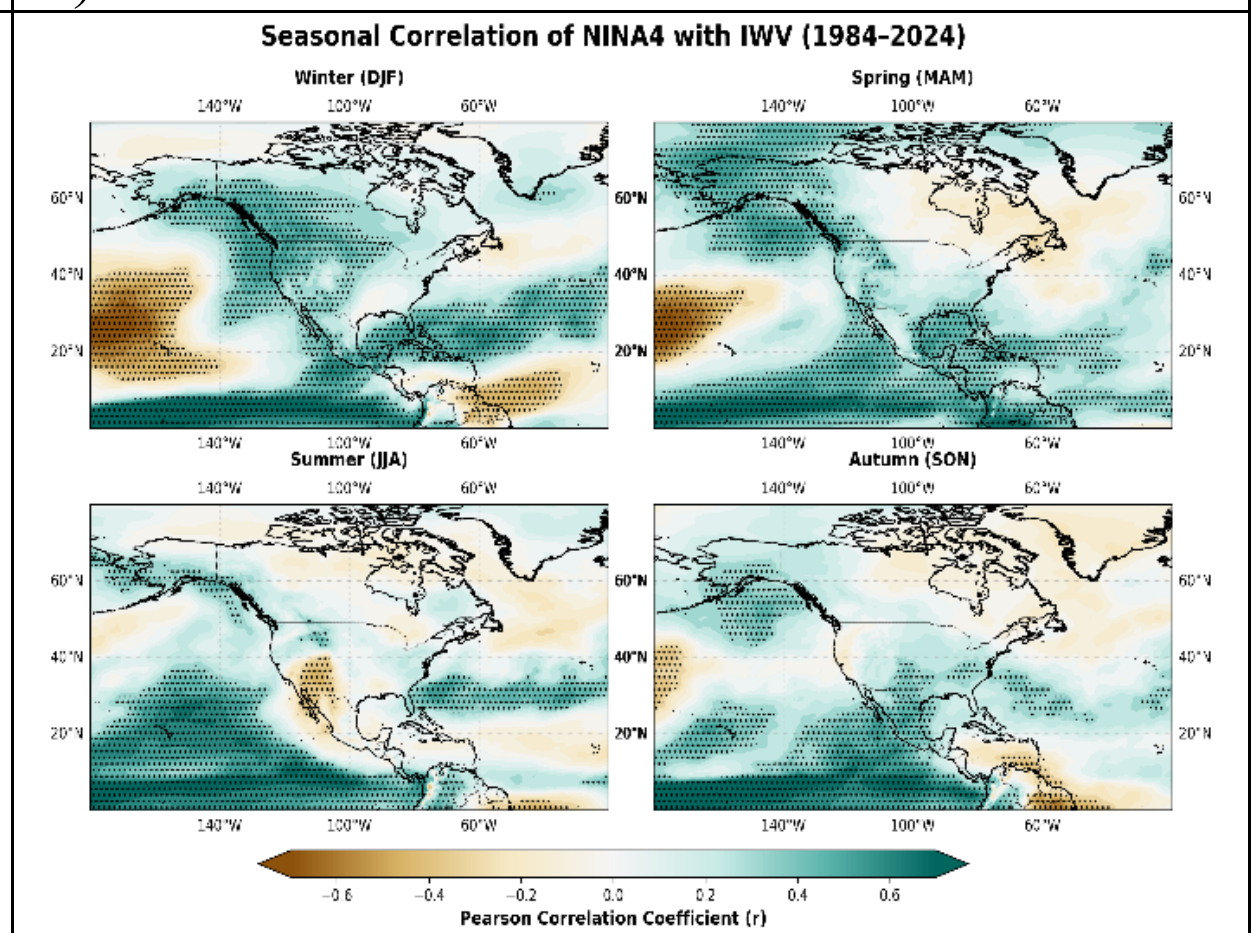
Seasonal Correlation of NINA4 with IWV (1984–2024)
Winter (DJF)
Spring (MAM)
Summer (JJA)
Autumn (SON)
Pearson Correlation Coefficient (r)

p)

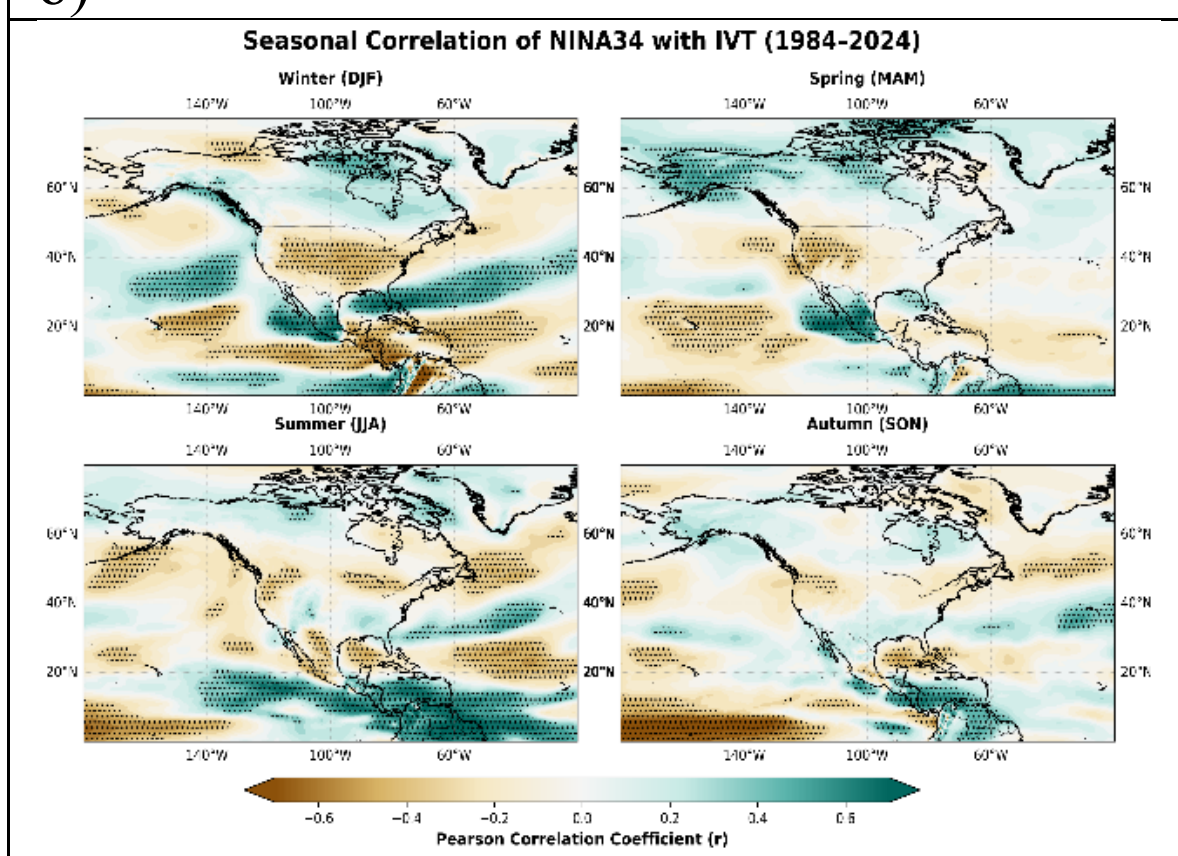
Seasonal Correlation of NINA34 with IVT (1984–2024)
Winter (DJF)
Spring (MAM)
Summer (JJA)
Autumn (SON)
Pearson Correlation Coefficient (r)

q)

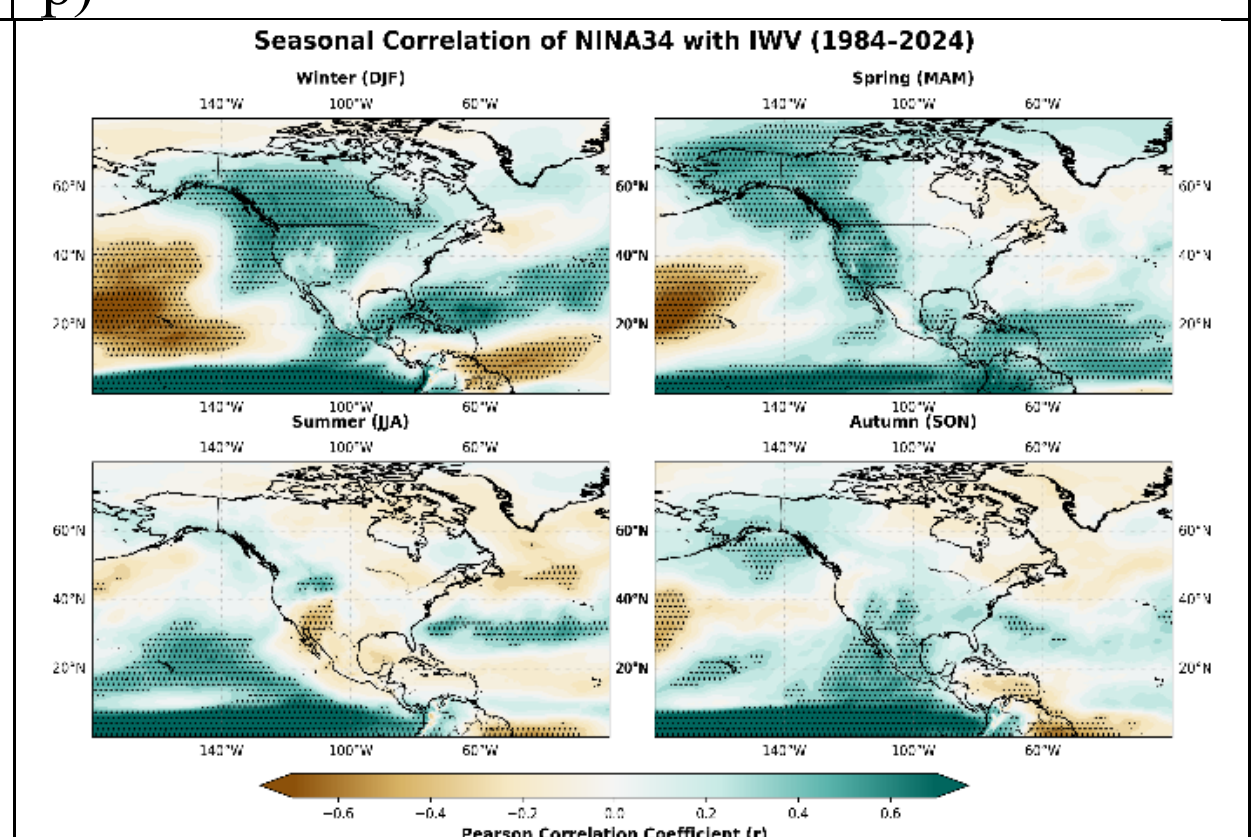
Seasonal Correlation of NINA34 with IWV (1984–2024)
Winter (DJF)
Spring (MAM)
Summer (JJA)
Autumn (SON)
Pearson Correlation Coefficient (r)

r)

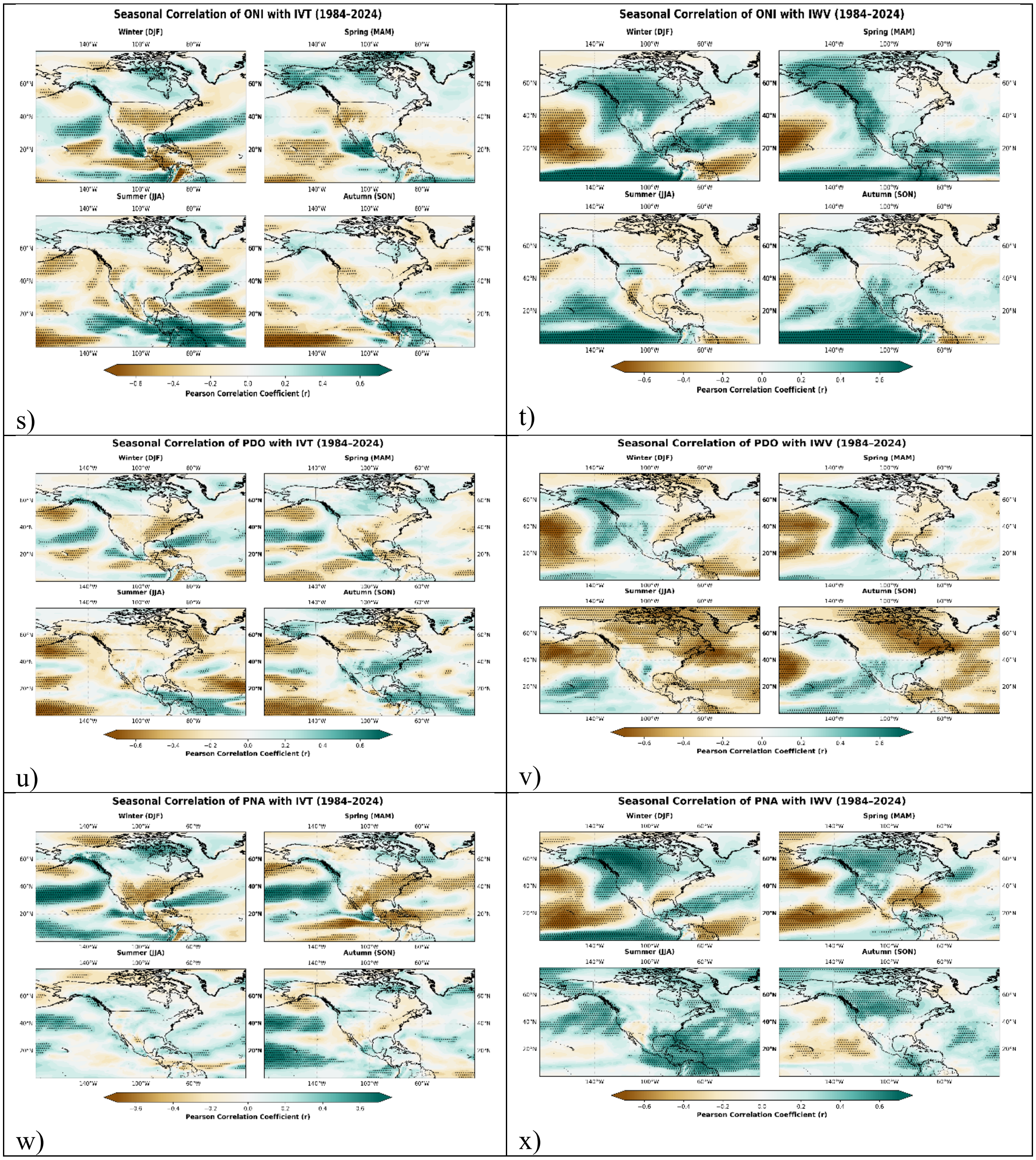

Fig. S1 Seasonal correlation maps between AR activity and climate teleconnections. Spatial distribution of seasonal correlations between AR activity, represented by the modified IVT and modified IWV indices, and twelve major climate teleconnection patterns during 1984–2024. The analyzed indices include the AO, NAO, PNA, PDO, ENSO phases (Niño 1+2, Niño 3, Niño 3.4, Niño 4, and Oceanic Niño indices ONI), MJO, CENSO, and MEIv2. Shading denotes the spatial magnitude of correlations between the modified IVT/IWV and each teleconnection, while black dots indicate areas that are statistically significant at the 95% confidence level. Collectively, these maps (a-x) reveal how both tropical and extratropical teleconnections modulate the variability and intensity of AR over NA.
